# Predicting Progression of Alzheimer’s Disease Using Ordinal Regression

**DOI:** 10.1371/journal.pone.0105542

**Published:** 2014-08-20

**Authors:** Orla M. Doyle, Eric Westman, Andre F. Marquand, Patrizia Mecocci, Bruno Vellas, Magda Tsolaki, Iwona Kłoszewska, Hilkka Soininen, Simon Lovestone, Steve C. R. Williams, Andrew Simmons

**Affiliations:** 1 Department of Neuroimaging, Institute of Psychiatry, King’s College London, London, United Kingdom; 2 Department of Neurobiology, Care Sciences and Society, Karolinska Institutet, Stockholm, Sweden; 3 NIHR Biomedical Research Centre for Mental Health at South London, London, United Kingdom; 4 NIHR Biomedical Research Unit for Dementia at South London, London, United Kingdom; 5 Institute of Gerontology and Geriatrics, University of Perugia, Perugia, Italy; 6 INSERM U 558, University of Toulouse, Toulouse, France; 7 Aristotle University of Thessaloniki, Thessaloniki, Greece; 8 Medical University of Lodz, Lodz, Poland; 9 University of Eastern Finland and University Hospital of Kuopio, Kuopio, Finland; 10 Maudsley NHS Foundation Trust, London, United Kingdom; 11 Institute of Psychiatry, King’s College London, London, United Kingdom; University of Manchester, United Kingdom

## Abstract

We propose a novel approach to predicting disease progression in Alzheimer’s disease (AD) – multivariate ordinal regression – which inherently models the ordered nature of brain atrophy spanning normal aging (CTL) to mild cognitive impairment (MCI) to AD. Ordinal regression provides probabilistic class predictions as well as a continuous index of disease progression – the ORCHID (Ordinal Regression Characteristic Index of Dementia) score. We applied ordinal regression to 1023 baseline structural MRI scans from two studies: the US-based Alzheimer’s Disease Neuroimaging Initiative (ADNI) and the European based AddNeuroMed program. Here, the acquired AddNeuroMed dataset was used as a completely independent test set for the ordinal regression model trained on the ADNI cohort providing an optimal assessment of model generalizability. Distinguishing CTL-like (CTL and stable MCI) from AD-like (MCI converters and AD) resulted in balanced accuracies of 82% (cross-validation) for ADNI and 79% (independent test set) for AddNeuroMed. For prediction of conversion from MCI to AD, balanced accuracies of 70% (AUC of 0.75) and 75% (AUC of 0.81) were achieved. The ORCHID score was computed for all subjects. We showed that this measure significantly correlated with MMSE at 12 months (ρ = –0.64, ADNI and ρ = –0.59, AddNeuroMed). Additionally, the ORCHID score can help fractionate subjects with unstable diagnoses (e.g. reverters and healthy controls who later progressed to MCI), moderately late converters (12–24 months) and late converters (24–36 months). A comparison with results in the literature and direct comparison with a binary classifier suggests that the performance of this framework is highly competitive.

## Introduction

Alzheimer’s disease (AD) is a neurodegenerative disorder characterized by progressive dementia that occurs in later life. In addition to devastating cognitive impairment, AD is characterized by progressive cerebral atrophy. The anatomical hallmarks of AD, cerebral atrophy and ventricular expansion, can be detected using magnetic resonance imaging (MRI). To model the spatial pattern of atrophy and predict conversion to AD, pattern recognition (PR) has been extensively applied, in particular to the Alzheimer’s disease neuroimaging initiative (ADNI) dataset [1]–[10]. Good performance has been well-established for discriminating healthy controls (CTL) and AD patients [11]. Therefore, most studies have focused on the more challenging problem of predicting conversion from mild cognitive impairment (MCI) to AD. The PR approach employed most commonly has been to train binary classifiers to discriminate CTL from AD, then apply these classifiers to make predictions for the MCI group [2], [6]–[9]. In these cases, the model is configured to discriminate between the extremities of disease progression while ignoring the intermediate states. A potential shortcoming of this approach is that it overlooks the ordinal nature of the disease progression: AD is associated with higher rates of brain atrophy than normal ageing with MCI between the two [12]–[14].

Here, we propose to use structural MRI acquired at baseline to predict disease progression across four clinical time points: CTL, MCI stable (MCI-s), MCI converter (MCI-c) and AD. To model these four groups *simultaneously* (distinct from pairwise comparisons) while considering the ordered relationship between the classes, we use multivariate ordinal regression [15]. Under this framework, the clinical groups are considered to lie on a continuum of disease progression, which provides a source of information that is not utilized by the conventional approach of producing predictions from models trained on the contrast between CTL (disease-free state) versus AD (disease state). We hypothesize that this method is therefore particularly well-suited to AD considering that brain atrophy/damage cannot be assumed to occur at a uniform rate [16]–[18]. The main advantages of performing multivariate ordinal regression over a mass univariate approach are that the method is able to make use of correlations between brain regions and provides predictions at a single subject level based on patterns in the data. In more detail, we have implemented multivariate ordinal regression using Gaussian processes in a Bayesian framework. This framework furnishes probabilistic predictions of which clinical time point (CTL, MCI-s, MCI-c or AD) a test case belongs too. In addition, to help alleviate the possibility of overfitting, this method implicitly regularises the solution via the prior over the parameters.

In this study, we explore the use of ordinal regression applied to baseline structural MRI data for automated early detection as well as diagnosis of AD using two large multicentre studies – the North American ADNI cohort and the European AddNeuroMed cohort. For the ADNI cohort, the performance of ordinal regression will be assessed using cross-validation with the AddNeuroMed cohort will be used as a completely independent test set. While cross-validation provides an estimate of the model generalizability; an independent test set provides the optimal way to assess the model generalizability [19]. In this case, the independent data set (AddNeuroMed) has been acquired so that it is compatible with ADNI but using different MRI scanners and in a European cohort.

## Materials and Methods

### Subjects

Subjects from two open access multicentre studies were used: AddNeuroMed (http://www.innomed-addneuromed.com) and ADNI (http://www.adni-info.org/). For AddNeuroMed follow-up is only available up to 12 months therefore, we only consider 12 month follow-up for the ADNI dataset for the main analysis. A total of 1023 subjects were included, 348 subjects from the AddNeuroMed study (119 AD, 119 MCI and 110 CTL, representing the entire sample) and 564 subjects from the ADNI study (147 AD, 226 MCI and 191 CTL; participant identifiers are listed in Table S1). For further validation, ADNI subjects with unstable diagnostic labels (reverters) and late converters (those who convert between 12–36 months) were selected using follow-up data to 36 months (4 AD, 89 MCI and 18 CTL; participant identifiers are listed in Table S2). Often these data have been previously discarded [1], [8], [20]. Here we propose to use these data as an additional test set to map these subjects onto the CTL to AD continuum. The subject characteristics are presented in Table 1. For more information about these datasets; specifically the inclusion/exclusion criteria and MRI acquisition see Text S1.

**Table 1:** Baseline subject characteristics.

	ADNI			ADNI Subset			AddNeuroMed		
	CTL	MCI	AD	CTL	MCI	AD	CTL	MCI	AD
N	191	226	147	18	89	4	110	119	119
Age	75.8±5.0	74.7±6.9	75.0±7.4	77.7±5.4	74.7±7.5	77.2±6.6	72.9±6.5	74.3±5.7	75.5±6.0
Female/Male	94/97	84/142	71/76	7/11	37/52	2/2	60/50	59/50	60/40
Education	16.1±2.8	15.8±2.9	14.8±3.0	16.6±2.6	15.6±3.0	13±3.8	10.8±4.8	8.9±4.3	8.0±4.0
MMSE	29.1±1.0	27.1±1.8	23.4±1.9	29.1±0.8	26.7±1.6	25.0±1.4	29.1±1.2	27.1±1.7	20.9±4.7
CDR-SOB	0.03±0.1	1.56±0.9	4.26±1.6	0.03±0.1	1.66±0.9	4.00±0.9	0.06±0.2	1.38±0.9	6.50±3.2
ApoE 4+ (%)	25.1	48.7	64.0	38.9	62.9	100	28.2	32.8	53.8

Data shown as mean ± standard deviation. Education is presented in years. ADNI subset refers to the group of reverters and later converters which were used for validation of the model but were not included in the training of the model. AD = Alzheimer’s disease, MCI = Mild Cognitive Impairment, CTL = healthy control, MMSE = Mini Mental State Examination. CDR-SOB – Clinical Dementia Rating – Sum Of Boxes.

The following diagnostic criteria were used:

**AD**: Mini mental state examination (MMSE) scores between 20–26, CDR of 0.5 or 1, and met NINCDS/ADRDA criteria for probable AD.
**MCI**: MMSE scores between 24–30, memory complaints with objective memory loss measured with Wechsler Memory Scale Logical Memory II (education adjusted scores), CDR of 0.5, absence of significant levels of impairment in other cognitive domains, preserved activities of daily leaving and absence of dementia.
**CTL**: MMSE scores between 24–30, CDR of 0, not depressed, non MCI, non-demented.

The subjects were re-assessed at several time points following baseline. Using the diagnostic criteria defined earlier and at the 12 month time point MCI subjects were divided into two groups: those that did not progress to AD (MCI-s) and those that progressed to AD (MCI-c). MCI subjects who converter after 12 months were not used to train the model but were used as a test validation set.

### Ethics Statement

We used ADNI subject data collected from 50 clinic sites. Ethics approval was obtained for each institution involved. The AddNeuroMed study was approved by ethical review boards in each participating country (local ethical review board at University of Perugia, University of Toulouse, Aristotle University of Thessaloniki, Medical University of Lodz, University of Eastern Finland and University Hospital of Kuopio and King’s College London). Both studies were conducted according to Good Clinical Practice guidelines, the Declaration of Helsinki, US 21CFR Part 50– Protection of Human Subjects, and Part 56– Institutional Review Boards, and pursuant to state and federal HIPAA regulations. Written consent was obtained where the research participant had capacity, and in those cases where dementia compromised capacity then assent from the patient and written consent from a relative, according to local law and process, was obtained. Consent was requested for data collection, sample storage and subsequent use of samples for research. The completed questionnaires were approved by each participating site’s Institutional Review Board. The data were anonymized before being shared.

### Regional volume segmentation and cortical thickness parcellation

For both studies, the imaging protocol included a high-resolution sagittal 3D T1-weighted Magnetization Prepared RApid Gradient Echo (MPRAGE) volume. Volumetric segmentation, cortical surface reconstruction and cortical parcellation, based on the FreeSurfer package (4.5.0, http://surfer.nmr.mgh.harvard.edu/), were used to quantify baseline thicknesses and volumes of brain regions, as detailed previously [21]. Sixty-eight cortical thickness measures (34 from each hemisphere) and 50 regional volumes were generated. Volumes of white matter hypointensities, optic chiasm, right and left vessel, and right and left choroid plexus were excluded. White matter hypointensities were excluded since most subjects were characterized by zero values. Volumetric measures were normalized by their intracranial volume while cortical thickness measures were not normalized [22]. Right and left measures were averaged [23]. In total this results in 57 measures to be used as input features for ordinal regression; 34 regional cortical thickness measures and 23 regional volumes (Table S3).

### Multivariate ordinal regression

Multivariate ordinal regression (ORGP) was performed using Gaussian processes in a Bayesian framework [15], [24], providing probabilistic predictions for class membership. The likelihood function captures the ordinal nature of the data using a soft threshold model. Crucially, these thresholds are learned from the training data to provide flexibility in the distances between classes. Additionally, the predictive mean of the latent function that models the ordinal continuum per test case (see Text S1) is used to measure the Ordinal Regression Characteristic Index of Dementia (ORCHID) whereby more positive values indicate a more AD-like brain structure and more negative values indicate a more CTL-like brain structure. To visualise the spatial pattern driving the discrimination multivariate maps can be constructed. This is achieved by visualising the weight vector to provide a spatial representation of the ordinal continuum.

#### Methodology of ordinal regression using Gaussian processes

Consider a training dataset 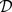 of N observations, 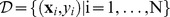 where each sample is a pair consisting of the input data vector 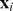 of dimension M and corresponding label 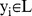 where L is a finite set of R ordinal categories, denoted 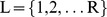. The column data vectors which in our case represent the cortical thickness and subcortical volumes for all N subjects are aggregated in the data matrix **X** with dimensions N×M. The targets are collected in vector y which represents the state of disease progression and are ranked from one to four whereby one: CTL, two: MCI-s, three: MCI-c and four: AD.

The main principle here is to assume an unobservable latent function 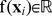 associated with each 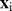 and assume a Gaussian process (GP) prior over 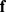, where 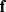 is a vector collecting all latent function values at the training points. The ordinal variable 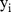 is dependent on the latent function 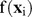 by modelling the ranks as intervals on the real line. This is achieved using a Bayesian framework. First, a GP prior 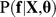 is placed on the latent function. The GP prior can be fully defined by a mean function 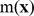 and a covariance function 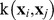. Here we define the GP as zero mean with a linear covariance matrix:
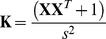
where 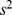 is a bias term that also controls the scaling of the latent function which in turn affects the variance of the predictive weights. We refer to this quantity as a hyperparameter, collected in the vector 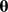, which will be optimised within this framework.

The joint probability of observing the ordinal variables, i.e. the likelihood is defined as
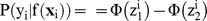
where 
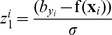
 and 
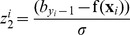
 with 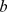 representing a threshold variable and 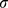 representing a noise term. 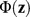 is the cumulative unit Gaussian whereby 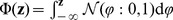. At the extremities of the ordinal continuum, the threshold variables serve as limits and are defined as 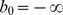 and 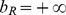, and the intermediate thresholds are further defined as 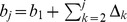 with positive padding variables 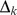 where k = 2,…,R–1. This formulation enforces ordinal constraints by dividing the real line into R contiguous intervals 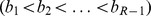 which map 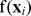 to the discrete variable 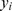. Note that the thresholds are not constrained to be equidistant.

Bayes theorem can be used to compute the posterior probability, hence enabling predictions to be made. The marginal likelihood, 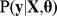 is known as the model evidence and is the metric used to learn the hyperparameters. To approximate the posterior distribution and model evidence we use the Laplace approximation at the maximum a posteriori (MAP) estimate [25]. Powell’s method is used to maximise the evidence and hence, infer the optimal hyperparameters [26].

Having set the hyperparameters, we now want to make predictions about a test case 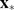 for which the target 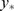 is unknown. Under the Laplace approximation, the predictive distribution for the latent function can be written as a Gaussian 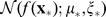 where the predictive mean and variance can be written as
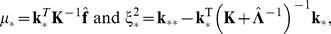
where 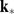 is the covariance between the test case and the training data, 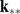 is the variance of the test case, 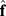 is the MAP estimate of the latent function, 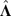 is a diagonal matrix whose ii-th entry is second derivative of the likelihood function training sample 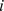 with respect to 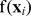.

The predictive distribution over the ordinal target 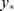 is
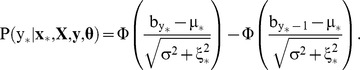



This distribution is used to assign the test case to an ordinal scale using
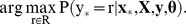



To visualise the spatial pattern driving the discrimination multivariate maps can be constructed. This is achieved by visualising the MAP estimate of the weight vector to provide a spatial representation of the decision boundary. This is analogous to the weight vector used for mapping SVM discrimination. For ORGP we can extract a vector 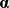, which is analogous to the weight vector in the function-space view of GP learning, whereby 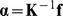. The maps are constructed by computing the posterior expectation 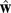 of the weight vector in the weight-space.
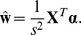



#### Implementation of ordinal regression

To assess the generalizability of the model two validation approaches were used. For ADNI data, the ordinal regression approach was embedded within a stratified 10-fold cross-validation scheme that preserves the relative frequencies of samples in each class. AddNeuroMed data were used as an independent validation set whereby the ordinal regression model was trained on the ADNI dataset and subsequently tested on the AddNeuroMed dataset.

To account for imbalanced subject numbers per class, the probabilistic predictions for each test case were recalibrated whereby the prediction per class was divided by the proportion of that class represented in the training set. The probabilistic predictions per test case and across all four classes were then re-normalised to sum to one [27].

This study aimed to predict the class label of each subject along an ordinal continuum representative of disease state. In addition, we aimed to develop a potentially useful tool for clinical decision-making and that can be easily compared with current approaches. For this purpose, we also consider the accuracy obtained when the ordinal labels were aggregated into two classes defined to reflect “CTL-like” (CTL or MCI-s) or “AD-like” (MCI-c or AD) subjects. Specifically, the prediction of CTL-like is considered correct if the subject belongs to the CTL or MCI-s groups and the prediction of AD-like is correct if the subject belongs to the MCI-c or AD group.

Multivariate maps were constructed to visualise the spatial pattern driving the ordinal regression. For Gaussian process models, this is achieved by visualising the maximum a posteriori estimate of the weight vector to provide a visualisation of the projection of the data ranging from CTL to AD (see Text S1 for more details). Since multivariate techniques are sensitive to spatial correlations across features (ROIs in this case), and the performance of the model is based on the entire pattern rather than individual regions, local inference should be avoided when interpreting these maps.

ORGP was implemented by the authors in MATLAB (The MathWorks, Natick, Massachusetts). Custom likelihood and inference scripts were written for compatibility with the GPML toolbox [25].

### Performance Metrics

The sensitivity and specificity of binary predictions were calculated for each pair. We report the balanced accuracy (mean of the sensitivity and specificity) which avoids inflated performance estimates for imbalanced datasets.

Confusion matrices and receiver operator characteristic (ROC) curves were used for visualisation. For the confusion matrices, the rows represent the true class labels and the columns represent the labels predicted by the learning machine. The diagonal elements represent correctly classified test cases whereas the off-diagonal elements represent misclassifications. An ROC curve involves plotting true positive rate (sensitivity) against the false positive rate (1-specificity) achieved by varying the threshold on the probabilities for a binary contrast. The area under this curve can also be used as a metric for assessing performance; an area of 1 implies perfect discrimination whereas as area of 0.5 implies random chance.

Differences in the distribution of the predictive mean from ORGP were assessed using a two sample Kolmogorov-Smirnov test; whereby p<0.05 implies that they are from different distributions. Spearman’s correlation was used to assess the relationship between ORCHID scores and MMSE scores.

## Results

Ordinal regression was applied to the four-class continuum spanning healthy controls (CTL), stable mild cognitive impairment (MCI-s), mild cognitive impairment with subsequent conversion (MCI-c) and Alzheimer’s disease (AD). The four class predictions were subsequently summarised to provide binary prediction of CTL-like or AD-like.

### Cross-validated ADNI results

**Figure 1:**
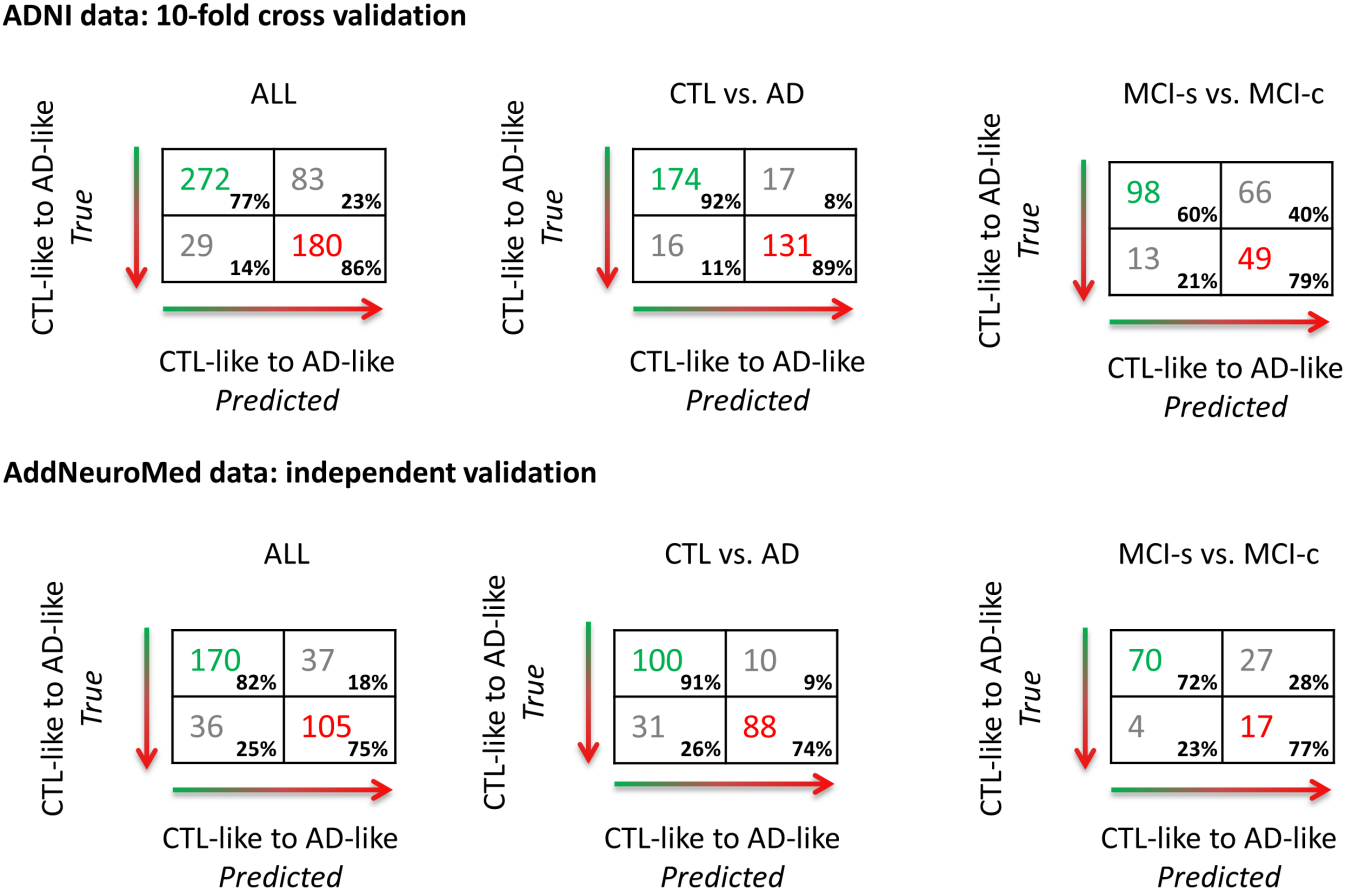
Confusion matrices for ordinal regression applied to two AD-related cohorts. The confusion matrix for the binarised CTL-like vs. AD-like (CTL and MCI-s vs. MCI-c and AD) is displayed on the left. For illustration purposes, on the right confusion matrices for two contrasts of interest: CTL vs. AD and MCI-s vs. MCI-c (note: training scheme is unchanged). The top panel displays the results achieved using 10-fold cross-validation on the ADNI dataset. The bottom panel displays the results achieved by applying the ordinal regression model trained on the ADNI dataset to the AddNeuroMed data set whereby the AddNeuroMed dataset represents an independent unseen validation of the performance of the method.

**Figure 2:**
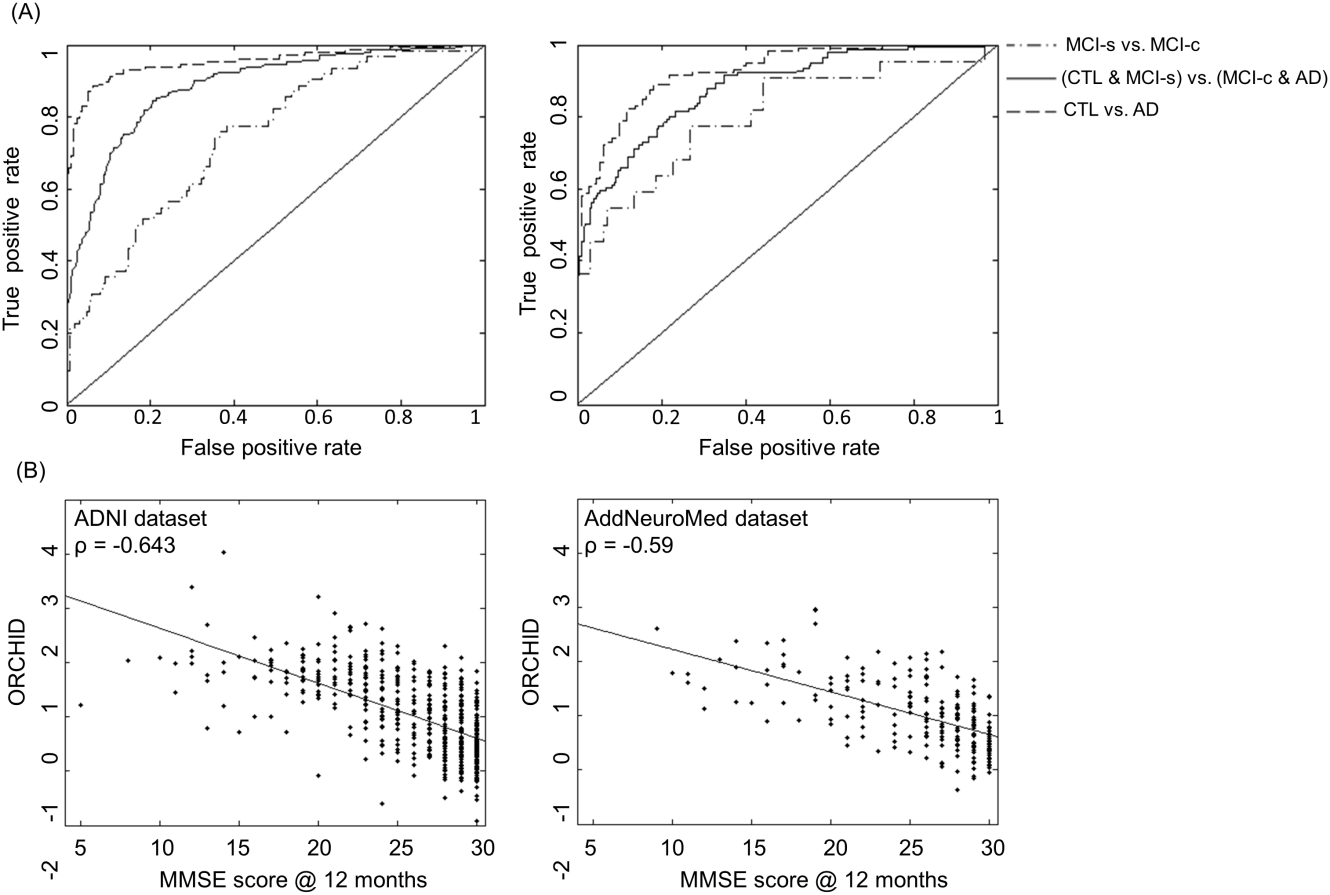
Performance curves and correlation plots for ordinal regression. (A): ROC curves for ordinal regression applied to the ADNI data set using 10-fold cross-validation (first panel) and using the AddNeuroMed data set as an independent test set. In both cases the ROC curves are shown from three contrasts. (B): Correlation plots of the MMSE score assessed at the 12 month follow-up against the Ordinal Regression Characteristic Index of Dementia (ORCHID) score for both the ADNI dataset and the AddNeuroMed dataset.

**Figure 3:**
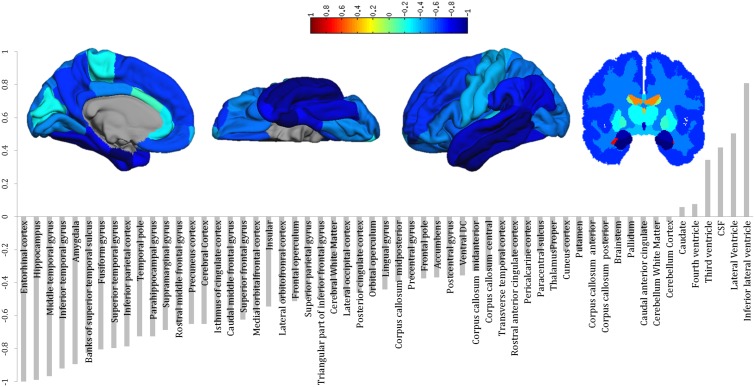
Multivariate discriminative weights computed using ordinal regression. For ordinal regression the weights can be interpreted as the projection of the data along the function space weight vector spanning CTL to MCI-s to MCI-s to AD. Note that the weights are symmetric across hemispheres. These weights are sensitive the spatial correlations in the data and therefore should not be interpreted in a univariate manner.

The performance of ordinal regression for the ADNI dataset using 10-fold cross-validation is presented in terms of three confusion matrices (Figure 1) and ROC curves (Figure 2(a)): the main contrast was CTL-like versus AD-like and two additional contrasts of interest were CTL vs. AD and MCI-s vs. MCI-c. For CTL-like versus AD-like, a balanced accuracy of 82% was achieved with an AUC of 0.88. Considering CTL versus AD, a balanced accuracy of 91% was achieved with an AUC of 0.95. For MCI-s versus MCI-c contrast a balanced accuracy of 70% and an AUC of 0.75 were found. The multivariate pattern of brain regions driving the discrimination is shown by Figure 3.

### Independent validation using AddNeuromed

To independently validate ordinal regression the model was trained on the ADNI data and tested on the AddNeuroMed data. The performance of the model is illustrated as confusion matrices (Figure 1) and ROC curves (Figure 2(a)). Comparing CTL and MCI-s to MCI-c and AD a balanced accuracy of 79% was obtained with an AUC of 0.88. Considering the CTL versus AD contrast a balanced accuracy of 83% was achieved with an AUC of 0.93. For MCI-c versus MCI-s a balanced accuracy of 75% with an AUC of 0.81 was obtained.

### Combined ADNI and AddNeuroMed Data

The performance of ordinal regression for the combined ADNI and AddNeuroMed datasets using stratified 10-fold cross-validation is presented in terms of three confusion matrices (Figure S2). Comparing CTL and MCI-s to MCI-c and AD a balanced accuracy of 82% was achieved with an AUC of 0.90. Considering the CTL versus AD contrast a balanced accuracy of 88% was achieved with an AUC of 0.95. For MCI-c versus MCI-s a balanced accuracy of 74% with an AUC of 0.80 were obtained.

### Correlation with MMSE at 12 months

The correlation between the ORCHID score and MMSE scores at 12 months was calculated (Figure 2(b)). For the ADNI dataset, a correlation coefficient of −0.643 (p<0.0001, Spearman’s ρ) was obtained following 10-fold cross-validation For the AddNeuroMed dataset, a correlation coefficient of −0.589 (p<0.0001, Spearman’s ρ) was obtained using this data as an independent test set.

### ADNI participants with unstable follow-up/late conversion

**Figure 4:**
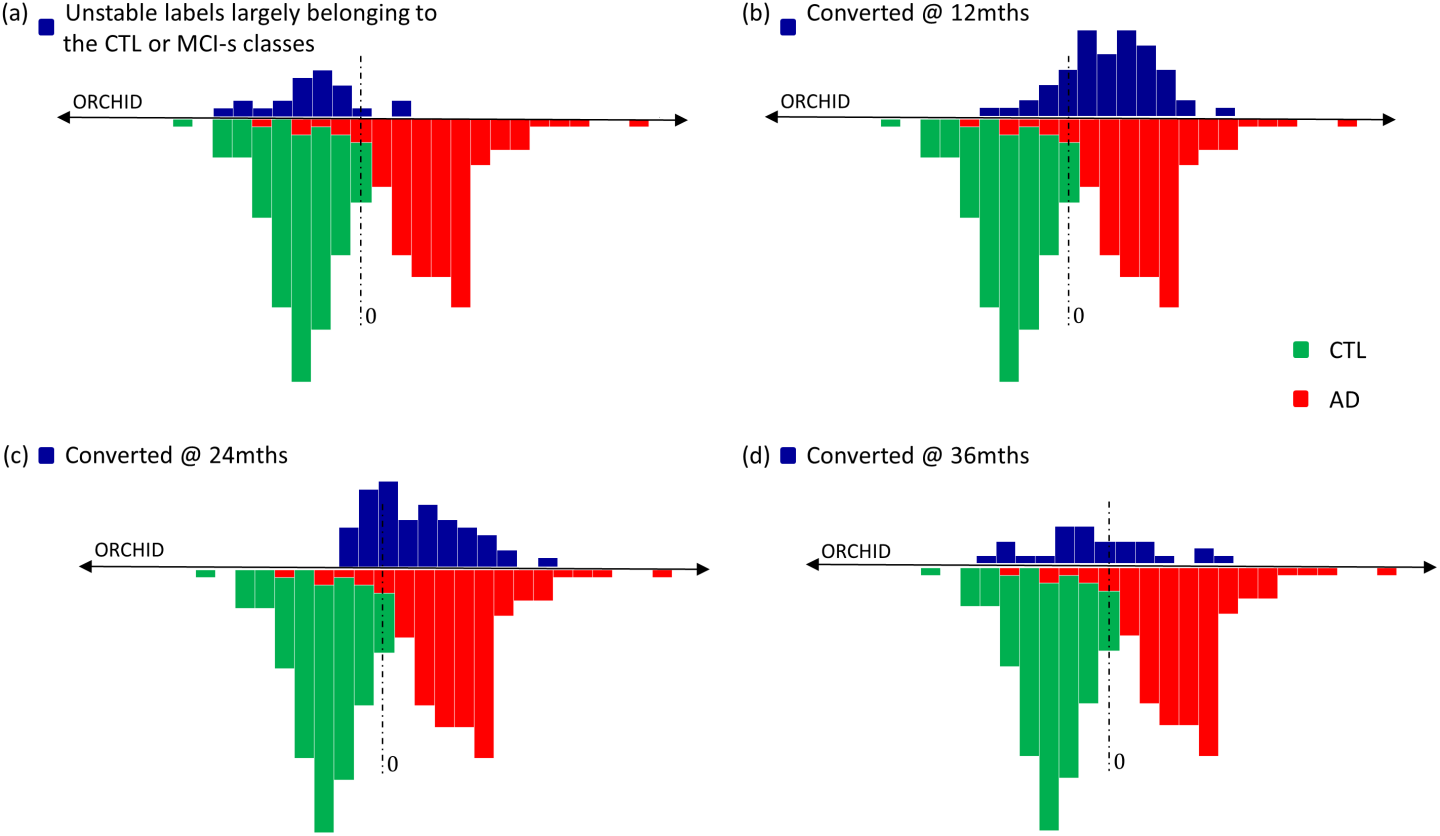
Distribution of the ORCHID (Ordinal Regression Characteristic Index of Dementia) score extracted from for the ADNI dataset representing the disease progressive continuum spanning CTL to MCI-s to MCI-c to AD. The lower portion of each plot represents the distributions for the CTL class (green) and the AD class (red). (a) represents the distribution of subjects with unstable labels across follow-ups, most of which appear to belong to either the CTL or MCI-s classes (N = 24). (b) represents the distribution of those who convert to AD by 12 month follow-up (N = 62) (i.e. MCI-c: the sample used for training and testing the ADNI-based ORGP model). (c) represents the distribution of those who convert to AD between the 12 and 24 month follow-up (N = 58). (d) represents the distribution of those who convert to AD between the 24 and 36 month follow-up (N = 29).

Ordinal regression trained on the ADNI dataset was tested using three subsets of the ADNI cohort: 1) **Subjects with unstable diagnosis** across follow-up time points including AD to CTL, MCI to CTL, AD to MCI and CTL subjects who progressed to MCI past 12 months. In their most recent follow-ups these subjects were labelled CTL or MCI-s. 2) **Moderately late converters**: subjects who convert to AD between the 12–24 month follow-up and 3) **Late converters**: subjects who convert to AD between the 24–36 month follow-up. In Figure 4, we map these subjects using the ORCHID score. For comparison, we also display distributions of the ORCHID scores for CTL and AD classes as well as MCI subjects who converted before 12 months. Subjects with unstable diagnoses overlap well with the CTL distribution, implying that these two distributions are similar (p>0.05, Kolmogorov-Smirnov test). For moderately late converters, the distribution is shifted towards AD with some overlap at the boundary. For the late converters, the distribution is more flat and spread across the continuum, implying these data are not accurately predicted by the model. The moderately late converters are likely to have been drawn from the same distribution as the converters at 12 months (p>0.05, Kolmogorov-Smirnov test) whereas the late converters are not drawn from the same sample (p = 0.00023, Kolmogorov-Smirnov test).

## Discussion

We have presented a novel application of ordinal regression which models structural brain changes as a continuum across healthy controls, stable MCI patients, MCI patients who convert to AD and AD patients. Conventionally, prediction of conversion to AD is achieved using binary classification trained on CTL and AD classes. Therefore, MCI classes are often not used to inform the discriminative model. We model all classes simultaneously as a continuum of disease progression. This is achieved using multivariate ordinal regression from which we can extract a probabilistic prediction of class membership as well as an index of AD progression – the ORCHID score. This method enables us to map subjects with unstable diagnosis (e.g. those who revert) and also those who are late converters to a continuum which is derived using all four classes as opposed to the extreme classes (CTL and AD).

A large body of literature exists on predicting conversion to AD from MCI [1]–[10]. Comparison between different studies in this literature is not trivial because of varying data types and feature construction, subject inclusion and exclusion criteria, criteria for determining conversion to AD, classification methods, classifier performance metrics, and most critically some papers that have not used independent training and test sets, ultimately resulting in biased (i.e. circular) analyses, as highlighted by Eskildsen et al. [3]. The contribution of this paper is a novel pattern recognition approach for AD diagnosis, therefore we have selected an appropriate subset of studies for comparison (i.e. having similar data selection heuristics and feature construction methods but varying pattern recognition techniques). The performance of ordinal regression in predicting conversion/stability at 12 months was 70% (AUC of 0.75) for the ADNI dataset. This is highly competitive considering recent studies. For example, Cuingnet et al. [1] achieved a balanced accuracy of 56% considering ROI-based cortical thickness (albeit excluding subcortical regions) and a balanced accuracy of 66% considering the volume of the hippocampus, both using support vector machine (SVM) classification. Using the same dataset as [1], Wolz et al. [10] achieved a balanced accuracy of 67% considering hippocampal volume only and linear discriminant classification. Eskildsen et al. [3] obtained comparable balanced accuracy (73%) and AUC (0.762) to the results reported here using cortical thickness measures and a linear discriminant classifier. However, these authors grouped subjects into time-homogenous groups of MCI converters, i.e. scans were selected 6, 12, 24 and 36 months prior to conversion. Using this rationale, 128 subjects fall into the 12 month converter group, which is approximately double the sample size used for training here (i.e. considering only the baseline data). Westman et al. [28] achieved a balanced accuracy of 63% using orthogonal partial least squares (OPLS) applied to measures of cortical thickness and subcortical volumes. Cui et al. [7] achieved a balanced accuracy of 63% using SVM on cortical thickness and subcortical volume features and considering follow-up to 24 months.

In order to optimally assess the generalizability of ordinal regression for predicting conversion to AD, we tested the model on a completely independent test set – the AddNeuroMed dataset. The balanced accuracy for predicting conversion/stability at 12 months was 75% (AUC of 0.81, sensitivity of 77% and specificity of 72%). Using an identical cohort and feature set to this study and OPLS, Westman et al. [28] obtained a balanced accuracy of 68% (sensitivity of 64%, specificity of 71%). The results reported by Westman et al. were obtained by training the OPLS classifier on the CTL and AD data from the combined AddNeuroMed and ADNI dataset and then tested on the MCI-s/-c classes from AddNeuroMed. In contrast, our results were obtained by applying ordinal regression to all four classes from the ADNI dataset and testing on the MCI-c/-s from AddNeuroMed. Given that both cohorts display similar patterns of brain atrophy [28], we suggest that the improvement in performance achieved here is likely to have been strongly driven by the use of ordinal regression to model brain atrophy as a continuum.

Fan et al. [4] published the first paper proposing a multivariate ranking approach for predicting conversion to AD. Fan et al. developed a pairwise ranking approach using binary classifiers with ordinal rules and presented their results using 4 class confusion matrices therefore, we can calculate the accuracy in the same manner as here. Using a subset of the ADNI dataset, a balanced accuracy of 54.2% was obtained for predicting conversion/stability. This is markedly lower than the performance obtained using our ordinal regression model which considers all classes simultaneously, albeit using a smaller sample and including later converters (>12months).

The spatial pattern of weights driving the ordinal regression model are presented in Figure 3. These weights are influenced by the value of either the cortical thickness or subcortical volume, the variance of the data and the parameterisation of the classifier. Therefore, a negative weight cannot be directly interpreted as an ROI displaying atrophy. Nonetheless we qualitatively note that the pattern of regions for ordinal regression is similar to discriminating regions for CTL vs. AD reported in previous studies [9], [28] with the entorhinal cortex, hippocampus and temporal lobe among the most negatively weighted regions and the ventricles and CSF among the most positively weighted regions.

Ordinal regression provides a single summary index of the state of disease progression - the ORCHID score. The rationale of the ORCHID score is similar to that of the SPARE-AD [2] and OPLS [29] indices. Specifically, a more positive ORCHID score implies a more AD-like brain structure and a more negative score implies a more CTL-like brain structure. The SPARE-AD study utilised a similar sized ADNI cohort, (170 MCI-s and 69 MCI-c) using CDR to define conversion; a balanced accuracy of 63% was obtained. For the OPLS index subjects from both the ADNI and AddNeuroMed datasets were used to achieve a balance accuracy of 68%. For comparison, we evaluated ordinal regression using both datasets and achieved a balanced accuracy of 74%. Both the SPARE-AD and OPLS indices are computed using the extremal ends of disease progression – CTL versus AD. In contrast, the ORCHID score developed here is derived from data across 4 states of disease progression (CTL to MCI-s to MCI-c to AD). Moreover, this method inherently models the ordinal nature of the states of disease progression which we infer from increasing levels of brain atrophy across the states. Ordinal regression also provides probabilistic predictions which enable us to recalibrate the predictions to account for the uneven number of subjects per class. Furthermore, ordinal regression was found to outperform binary classification using Gaussian processes [25], [30] (4% increase for ADNI, 6% increase for AddNeuroMed, Figure S1) trained in a similar manner to SPARE-AD and OPLS whereby the CTL versus AD subjects from ADNI were used for training and MCI-s/c subjects from AddNeuroMed and ADNI were used for testing (see Text S1).

The ORCHID score was also found to be significantly correlated with the MMSE score at 12 month follow-up for both the ADNI dataset ρ = −0.64 (cross-validation) and the AddNeuroMed dataset ρ = −0.59 (independent validation). Stonnington et al. [31] investigated the utility of baseline MRI for directly regressing *baseline* MMSE scores on structural imaging data. Using a similar sized ADNI cohort they achieved a correlation coefficient of ρ = 0.48. The magnitude of the correlation coefficient reported here is higher while also being compared to the MMSE at 12 month follow-up rather than baseline.

We employed the ORCHID score to help fractionate subjects with unstable diagnoses (e.g. reverters and healthy controls who later progressed to MCI), moderately late converters (12–24 months) and late converters (24–36 months). We found the distribution of the predictive mean for the unstable diagnosis group was similar to the distribution for healthy controls. We consider this to be appropriate given that most people in this group had reverted from a diagnosis of AD or MCI-c to CTL or MCI-s. The moderately late converters were found to have similar distribution to the 12 month converters. However, the late converters were found to have a significantly different distribution of predictive means. Qualitatively, the predictive means are spread across the continuum (Figure 4). This implies that late converters are more difficult to characterise which may be as at baseline they are at an early stage of disease progression in terms of brain pathology. This is in keeping with other studies which found that predictive performance was highest closer to the point of conversion [3], [32]. Adaszewsk et al. [32] proposed that atrophy is restricted to a few brain regions in the early stages of disease progression as atrophy in AD is assumed to follow the spreading pattern of beta amyloid of tau depositions with a delay of several years,. Therefore, the pattern of brain changes for late converters (24 to 36 months) may be distinct from the pattern of earlier converters and those already diagnosed with AD and hence this may explain why late converters are not well-characterised by the ORCHID score.

We have presented a probabilistic multivariate framework for ordinal regression. A comparison with results in the literature and direct comparison with a binary classifier suggests that the performance of this framework is highly competitive. To further explore the performance of this technique, it may be interesting to extend this framework to the multi-modal case incorporating multiple data sources. This may provide further improved performance if complimentary information is spread across data sources.

Methodologically, this technique offers several advantages: the inherent ordinal nature of the disease progression of AD is captured and two quantitative predictions are provided which can be readily interpreted. The first is a probabilistic prediction at the individual subject level, which indicates the categorical group membership (e.g. CTL-like or AD-like) and can be directly applied to assist clinical decision making, following appropriate validation. Moreover, the method quantifies the certainty of this prediction, which is equally crucial for clinical applications, for example to account for class imbalance [27]. The second type of prediction is the ORCHID score, which quantifies disease progression providing a visualisation of where an individual subject lies on a continuum spanning healthy to disease state. Overall, we propose that multivariate ordinal regression is a potentially powerful method for identifying those at risk of progressing to AD.

## Supporting Information

Figure S1 **Confusion matrices obtained for MCI stable versus converters from the ADNI and AddNeuroMed cohorts using a binary Gaussian process classification trained on CTL versus AD subjects from the ADNI cohort.**
(TIF)

Figure S2 **Confusion matrices for ordinal regression applied to the combined data from ADNI and AddNeuroMed using 10-fold cross validation.** The confusion matrix for the binarised CTL-like vs. AD-like (CTL and MCI-s vs. MCI-c and AD) is displayed on the left. For illustration purposes, on the right confusion matrices for two contrasts of interest: CTL vs. AD and MCI-s vs. MCI-c (note: training scheme is unchanged).
(TIF)

Table S1 **List of participants selected from the ADNI dataset for training and testing the ordinal regression model.**
(DOCX)

Table S2 **List of participants selected from the ADNI dataset for validating the ordinal regression model.**
(DOCX)

Table S3 **Variables included in the ordinal regression analysis.** 57 variables in total, 34 cortical thickness measures and 23 volumetric measures.
(DOCX)

Text S1 (DOCX)
