# Supplementary figures and images for: Predicting Progression of Alzheimer’s Disease Using Ordinal Regression
Source: PLoS One. 2014 Aug 20;9(8):e105542. doi: 10.1371/journal.pone.0105542 (PMC4139338; doi:10.1371/journal.pone.0105542)

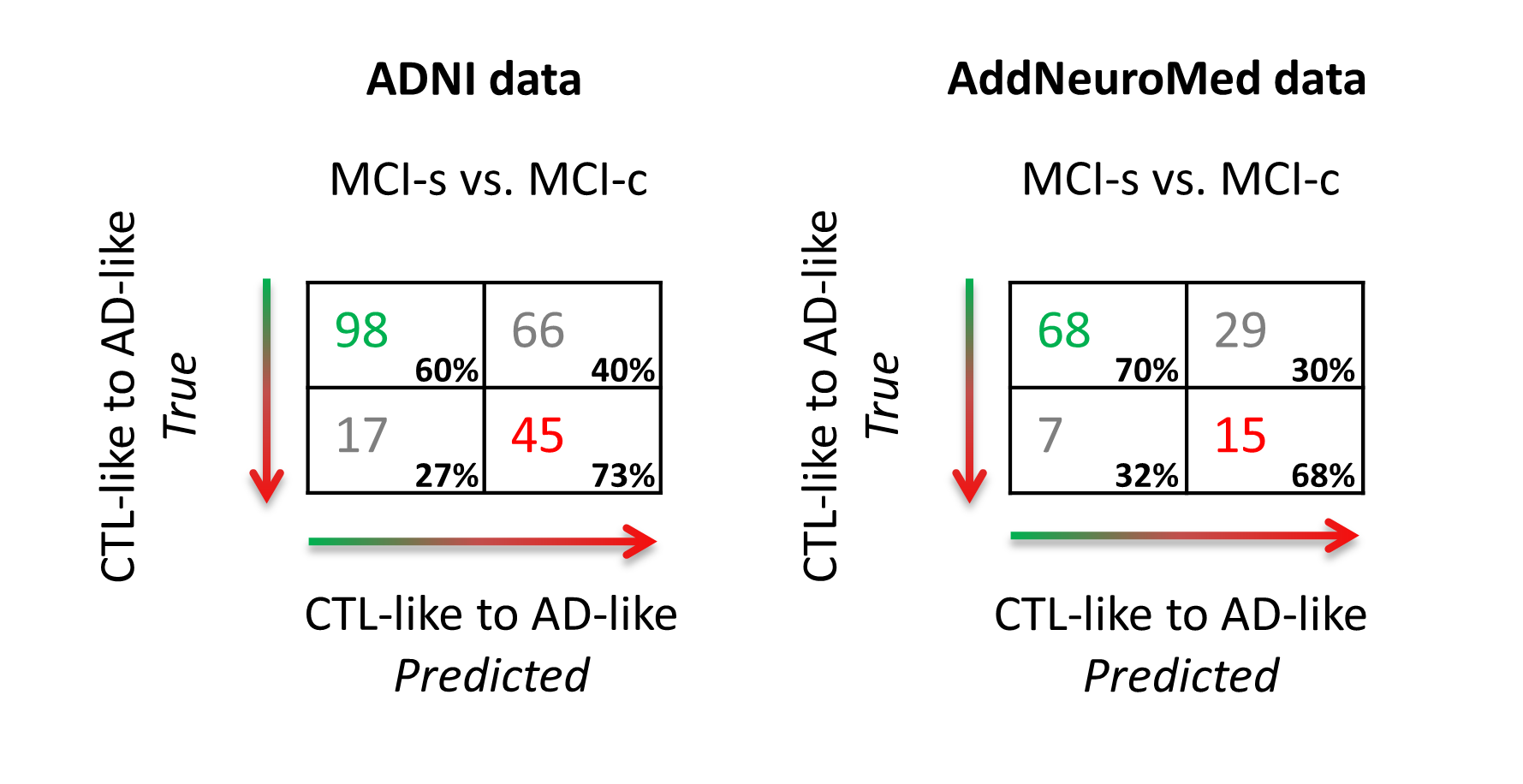

Supplement: Figure S1 — Confusion matrices obtained for MCI stable versus converters from the ADNI and AddNeuroMed cohorts using a binary Gaussian process classification trained on CTL versus AD subjects from the ADNI cohort. (TIF) [file pone.0105542.s001.tif]

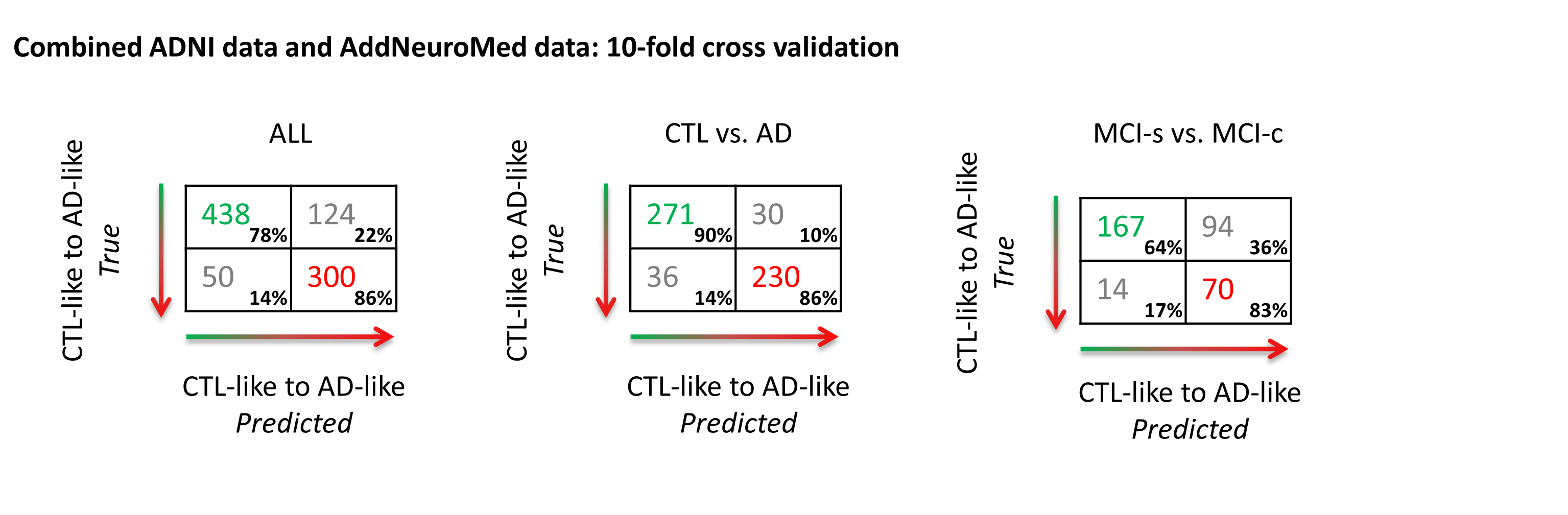

Supplement: Figure S2 — Confusion matrices for ordinal regression applied to the combined data from ADNI and AddNeuroMed using 10-fold cross validation. The confusion matrix for the binarised CTL-like vs. AD-like (CTL and MCI-s vs. MCI-c and AD) is displayed on the left. For illustration purposes, on the right confusion matrices for two contrasts of interest: CTL vs. AD and MCI-s vs. MCI-c (note: training scheme is unchanged). (TIF) [file pone.0105542.s002.tif]
