# Supplementary material for: Predicting Progression of Alzheimer’s Disease Using Ordinal Regression
Source: PLoS One. 2014 Aug 20;9(8):e105542. doi: 10.1371/journal.pone.0105542 (PMC4139338; doi:10.1371/journal.pone.0105542)
Supplement: Table S1 — List of participants selected from the ADNI dataset for training and testing the ordinal regression model. (DOCX) [file pone.0105542.s003.docx]

| 002_S_0295 | 011_S_1282 | 022_S_0129 | 032_S_0479 | 051_S_1072 | 082_S_1119 | 114_S_1106 | 130_S_0285 |
| --- | --- | --- | --- | --- | --- | --- | --- |
| 002_S_0413 | 012_S_0634 | 022_S_0130 | 032_S_0677 | 051_S_1123 | 082_S_1256 | 114_S_1118 | 130_S_0423 |
| 002_S_0559 | 012_S_0637 | 022_S_0543 | 032_S_0718 | 051_S_1131 | 082_S_1377 | 116_S_0361 | 130_S_0449 |
| 002_S_0619 | 012_S_0689 | 022_S_0544 | 032_S_1101 | 051_S_1296 | 094_S_0434 | 116_S_0370 | 130_S_0505 |
| 002_S_0685 | 012_S_0712 | 022_S_0961 | 032_S_1169 | 051_S_1331 | 094_S_0526 | 116_S_0382 | 130_S_0783 |
| 002_S_0729 | 012_S_0720 | 022_S_1097 | 033_S_0516 | 052_S_0671 | 094_S_0531 | 116_S_0392 | 130_S_0886 |
| 002_S_0782 | 012_S_0803 | 022_S_1394 | 033_S_0567 | 052_S_0951 | 094_S_0692 | 116_S_0487 | 130_S_0956 |
| 002_S_0816 | 012_S_0932 | 023_S_0031 | 033_S_0723 | 052_S_1054 | 094_S_0711 | 116_S_0648 | 130_S_0969 |
| 002_S_0938 | 012_S_1033 | 023_S_0042 | 033_S_0724 | 052_S_1250 | 094_S_0921 | 116_S_0657 | 130_S_1201 |
| 002_S_0954 | 012_S_1133 | 023_S_0058 | 033_S_0733 | 052_S_1251 | 094_S_1027 | 116_S_0752 | 130_S_1290 |
| 002_S_1018 | 012_S_1165 | 023_S_0061 | 033_S_0734 | 052_S_1346 | 094_S_1090 | 116_S_0834 | 130_S_1337 |
| 002_S_1155 | 012_S_1212 | 023_S_0078 | 033_S_0739 | 053_S_0389 | 094_S_1102 | 116_S_1232 | 131_S_0123 |
| 002_S_1261 | 012_S_1292 | 023_S_0081 | 033_S_0741 | 053_S_0507 | 094_S_1164 | 116_S_1249 | 131_S_0319 |
| 002_S_1268 | 012_S_1321 | 023_S_0083 | 033_S_0889 | 053_S_0621 | 094_S_1267 | 121_S_1322 | 131_S_0441 |
| 002_S_1280 | 013_S_0502 | 023_S_0084 | 033_S_0920 | 053_S_0919 | 094_S_1293 | 121_S_1350 | 131_S_0457 |
| 003_S_0907 | 013_S_0575 | 023_S_0093 | 033_S_0922 | 053_S_1044 | 094_S_1314 | 123_S_0072 | 131_S_0497 |
| 003_S_0908 | 013_S_0860 | 023_S_0139 | 033_S_0923 | 057_S_0464 | 094_S_1330 | 123_S_0088 | 131_S_0691 |
| 003_S_0931 | 013_S_0996 | 023_S_0376 | 033_S_1016 | 057_S_0474 | 094_S_1397 | 123_S_0091 | 131_S_1301 |
| 003_S_0981 | 013_S_1035 | 023_S_0855 | 033_S_1086 | 057_S_0643 | 094_S_1398 | 123_S_0094 | 132_S_0987 |
| 003_S_1074 | 013_S_1120 | 023_S_0916 | 033_S_1098 | 057_S_0818 | 094_S_1402 | 123_S_0106 | 133_S_0433 |
| 003_S_1122 | 013_S_1161 | 023_S_0926 | 033_S_1116 | 057_S_0934 | 094_S_1417 | 123_S_0113 | 133_S_0488 |
| 005_S_0221 | 013_S_1186 | 023_S_0963 | 033_S_1279 | 057_S_0941 | 098_S_0149 | 123_S_0162 | 133_S_0493 |
| 005_S_0222 | 013_S_1205 | 023_S_1046 | 033_S_1281 | 057_S_1217 | 098_S_0160 | 123_S_0298 | 133_S_0525 |
| 005_S_0324 | 013_S_1275 | 023_S_1126 | 033_S_1284 | 057_S_1269 | 098_S_0171 | 123_S_1300 | 133_S_0629 |
| 005_S_0448 | 013_S_1276 | 023_S_1190 | 033_S_1285 | 057_S_1371 | 098_S_0269 | 126_S_0605 | 133_S_0638 |
| 005_S_0546 | 014_S_0169 | 023_S_1247 | 033_S_1308 | 057_S_1373 | 098_S_0896 | 126_S_0606 | 133_S_0771 |
| 005_S_0553 | 014_S_0328 | 023_S_1262 | 033_S_1309 | 062_S_0535 | 099_S_0040 | 126_S_0680 | 133_S_0792 |
| 005_S_0572 | 014_S_0519 | 024_S_0985 | 035_S_0048 | 062_S_0578 | 099_S_0051 | 126_S_0709 | 133_S_0912 |
| 005_S_0602 | 014_S_0520 | 024_S_1171 | 035_S_0156 | 062_S_0690 | 099_S_0054 | 126_S_0784 | 133_S_0913 |
| 005_S_0610 | 014_S_0557 | 024_S_1307 | 035_S_0204 | 062_S_0730 | 099_S_0060 | 126_S_0891 | 133_S_1031 |
| 005_S_0814 | 014_S_0558 | 027_S_0074 | 035_S_0292 | 062_S_0768 | 099_S_0090 | 126_S_1187 | 133_S_1055 |
| 005_S_0929 | 014_S_0658 | 027_S_0116 | 035_S_0341 | 062_S_0793 | 099_S_0111 | 126_S_1221 | 133_S_1170 |
| 005_S_1224 | 014_S_1095 | 027_S_0118 | 035_S_0555 | 062_S_1099 | 099_S_0291 | 127_S_0259 | 136_S_0086 |
| 005_S_1341 | 016_S_0354 | 027_S_0120 | 036_S_0576 | 062_S_1182 | 099_S_0352 | 127_S_0260 | 136_S_0107 |
| 006_S_0498 | 016_S_0359 | 027_S_0256 | 036_S_0577 | 067_S_0019 | 099_S_0372 | 127_S_0393 | 136_S_0184 |
| 006_S_0547 | 016_S_0538 | 027_S_0307 | 036_S_0656 | 067_S_0029 | 099_S_0470 | 127_S_0394 | 136_S_0186 |
| 006_S_0681 | 016_S_0991 | 027_S_0403 | 036_S_0672 | 067_S_0038 | 099_S_0533 | 127_S_0431 | 136_S_0194 |
| 006_S_0731 | 016_S_1028 | 027_S_0404 | 036_S_0673 | 067_S_0056 | 099_S_0534 | 127_S_0622 | 136_S_0196 |
| 007_S_0068 | 016_S_1117 | 027_S_0408 | 036_S_0748 | 067_S_0059 | 099_S_1034 | 127_S_0684 | 136_S_0299 |
| 007_S_0070 | 018_S_0043 | 027_S_0417 | 036_S_0759 | 067_S_0076 | 099_S_1144 | 127_S_0754 | 136_S_0300 |
| 007_S_0249 | 018_S_0080 | 027_S_0461 | 036_S_0760 | 067_S_0077 | 100_S_0006 | 127_S_0844 | 136_S_0579 |
| 007_S_0316 | 018_S_0087 | 027_S_0485 | 036_S_0813 | 067_S_0110 | 100_S_0015 | 127_S_0925 | 136_S_0695 |
| 007_S_0344 | 018_S_0142 | 027_S_0644 | 036_S_0945 | 067_S_0176 | 100_S_0035 | 127_S_1140 | 136_S_1227 |
| 007_S_0414 | 018_S_0286 | 027_S_0850 | 036_S_1001 | 067_S_0177 | 100_S_0047 | 127_S_1419 | 137_S_0158 |
| 007_S_0698 | 018_S_0335 | 027_S_1045 | 036_S_1023 | 067_S_0243 | 100_S_0069 | 127_S_1427 | 137_S_0283 |
| 007_S_1206 | 018_S_0369 | 027_S_1081 | 037_S_0150 | 067_S_0257 | 100_S_0190 | 128_S_0135 | 137_S_0301 |
| 007_S_1222 | 018_S_0425 | 027_S_1082 | 037_S_0303 | 067_S_0284 | 100_S_0296 | 128_S_0200 | 137_S_0366 |
| 007_S_1304 | 018_S_0633 | 027_S_1213 | 037_S_0327 | 067_S_0290 | 100_S_0747 | 128_S_0216 | 137_S_0459 |
| 007_S_1339 | 018_S_0682 | 027_S_1254 | 037_S_0377 | 067_S_0336 | 100_S_0930 | 128_S_0225 | 137_S_0481 |
| 009_S_0751 | 020_S_0097 | 027_S_1385 | 037_S_0454 | 067_S_0607 | 100_S_0995 | 128_S_0229 | 137_S_0631 |
| 009_S_0842 | 020_S_0213 | 029_S_0824 | 037_S_0467 | 067_S_0812 | 100_S_1062 | 128_S_0272 | 137_S_0668 |
| 009_S_0862 | 020_S_1288 | 029_S_0836 | 037_S_0501 | 067_S_1253 | 100_S_1226 | 128_S_0310 | 137_S_0686 |
| 009_S_1030 | 021_S_0141 | 029_S_0866 | 037_S_0552 | 068_S_0109 | 100_S_1286 | 128_S_0500 | 137_S_0796 |
| 010_S_0067 | 021_S_0159 | 029_S_0914 | 037_S_0588 | 068_S_0127 | 109_S_0950 | 128_S_0517 | 137_S_0800 |
| 010_S_0419 | 021_S_0273 | 029_S_0999 | 037_S_0627 | 068_S_0210 | 109_S_0967 | 128_S_0522 | 137_S_0825 |
| 010_S_0420 | 021_S_0276 | 029_S_1056 | 037_S_1078 | 068_S_0442 | 109_S_1014 | 128_S_0528 | 137_S_0972 |
| 010_S_0472 | 021_S_0332 | 029_S_1215 | 037_S_1225 | 068_S_0473 | 109_S_1114 | 128_S_0545 | 137_S_0994 |
| 010_S_0786 | 021_S_0337 | 029_S_1218 | 037_S_1421 | 068_S_0872 | 109_S_1157 | 128_S_0608 | 137_S_1041 |
| 010_S_0829 | 021_S_0343 | 029_S_1318 | 041_S_0125 | 068_S_1075 | 109_S_1183 | 128_S_0715 | 137_S_1414 |
| 011_S_0002 | 021_S_0424 | 029_S_1384 | 041_S_0262 | 072_S_0315 | 109_S_1343 | 128_S_0740 | 141_S_0717 |
| 011_S_0005 | 021_S_0626 | 031_S_0321 | 041_S_0282 | 073_S_0089 | 114_S_0166 | 128_S_0770 | 141_S_0726 |
| 011_S_0008 | 021_S_0642 | 031_S_0351 | 041_S_0446 | 073_S_0311 | 114_S_0173 | 128_S_0863 | 141_S_0851 |
| 011_S_0010 | 021_S_0647 | 031_S_0554 | 041_S_0549 | 073_S_0312 | 114_S_0374 | 128_S_1088 | 141_S_0852 |
| 011_S_0016 | 021_S_0753 | 031_S_0568 | 041_S_0598 | 073_S_0386 | 114_S_0378 | 128_S_1242 | 141_S_0915 |
| 011_S_0021 | 021_S_0984 | 031_S_0618 | 041_S_0679 | 073_S_0565 | 114_S_0410 | 128_S_1406 | 141_S_0982 |
| 011_S_0023 | 021_S_1109 | 031_S_0830 | 041_S_1002 | 073_S_0746 | 114_S_0416 | 128_S_1409 | 141_S_1004 |
| 011_S_0053 | 022_S_0004 | 031_S_0867 | 041_S_1260 | 082_S_0304 | 114_S_0458 | 128_S_1430 | 141_S_1137 |
| 011_S_0183 | 022_S_0007 | 031_S_1066 | 041_S_1368 | 082_S_0363 | 114_S_0601 | 129_S_0778 | 141_S_1255 |
| 011_S_0241 | 022_S_0014 | 031_S_1209 | 041_S_1412 | 082_S_0832 | 114_S_0979 | 129_S_1246 | 941_S_1194 |
| 011_S_0856 | 022_S_0066 | 032_S_0147 | 041_S_1418 | 082_S_1079 | 114_S_1103 | 130_S_0102 | 941_S_1195 |
| 011_S_1080 | 022_S_0096 | 032_S_0400 | 041_S_1423 |  |  |  |  |
| Table S1 List of participants selected from the ADNI dataset for training and testing the ordinal regression model. | | | | | | | |
