# Supplementary material for: Predicting Progression of Alzheimer’s Disease Using Ordinal Regression
Source: PLoS One. 2014 Aug 20;9(8):e105542. doi: 10.1371/journal.pone.0105542 (PMC4139338; doi:10.1371/journal.pone.0105542)
Supplement: Table S2 — List of participants selected from the ADNI dataset for validating the ordinal regression model. (DOCX) [file pone.0105542.s004.docx]

| 002_S_0955 | 011_S_0362 | 022_S_0219 | 032_S_0187 | 041_S_1435 | 098_S_0884 | 126_S_0708 | 133_S_0727 |
| --- | --- | --- | --- | --- | --- | --- | --- |
| 002_S_1070 | 011_S_0861 | 022_S_0750 | 032_S_0214 | 052_S_0952 | 099_S_0492 | 126_S_0865 | 136_S_0429 |
| 003_S_1021 | 012_S_1009 | 022_S_1351 | 032_S_0978 | 052_S_1352 | 100_S_0892 | 128_S_0138 | 136_S_0873 |
| 003_S_1057 | 013_S_0240 | 023_S_0126 | 032_S_1037 | 057_S_0779 | 100_S_1113 | 128_S_0167 | 136_S_0874 |
| 005_S_0223 | 013_S_0325 | 023_S_0217 | 033_S_0511 | 057_S_0839 | 109_S_0777 | 128_S_0188 | 137_S_0438 |
| 006_S_0484 | 013_S_0592 | 023_S_0331 | 033_S_0513 | 057_S_1007 | 109_S_0876 | 128_S_0227 | 137_S_0841 |
| 006_S_0653 | 014_S_0356 | 023_S_0388 | 033_S_0725 | 057_S_1265 | 109_S_1013 | 128_S_0230 | 137_S_0973 |
| 006_S_0675 | 014_S_0548 | 023_S_0604 | 033_S_0906 | 062_S_1299 | 109_S_1192 | 128_S_0245 | 141_S_0696 |
| 006_S_1130 | 014_S_0563 | 023_S_0887 | 035_S_0997 | 067_S_0098 | 114_S_0228 | 128_S_0258 | 141_S_0767 |
| 007_S_0041 | 016_S_0702 | 023_S_1289 | 036_S_0869 | 067_S_0828 | 116_S_0360 | 128_S_0611 | 141_S_0810 |
| 007_S_0101 | 016_S_0769 | 023_S_1306 | 036_S_0976 | 067_S_1185 | 116_S_0649 | 128_S_0947 | 141_S_0853 |
| 007_S_0128 | 016_S_1121 | 024_S_1063 | 036_S_1135 | 068_S_1191 | 116_S_1083 | 128_S_1043 | 141_S_1024 |
| 007_S_0293 | 016_S_1263 | 024_S_1393 | 036_S_1240 | 073_S_0518 | 116_S_1243 | 128_S_1148 | 141_S_1052 |
| 007_S_1248 | 018_S_0155 | 027_S_0179 | 037_S_0539 | 073_S_0909 | 116_S_1271 | 128_S_1408 | 141_S_1094 |
| 009_S_1334 | 018_S_0406 | 027_S_0835 | 037_S_0566 | 082_S_0640 | 116_S_1315 | 130_S_0232 | 141_S_1152 |
| 009_S_1354 | 018_S_0450 | 029_S_0843 | 041_S_0314 | 082_S_0761 | 123_S_0108 | 130_S_0289 | 141_S_1378 |
| 010_S_0904 | 020_S_0883 | 029_S_0878 | 041_S_0898 | 094_S_0489 | 123_S_0390 | 130_S_1200 | 941_S_1197 |
| 011_S_0022 | 020_S_0899 | 029_S_1073 | 041_S_1391 | 094_S_1241 | 126_S_0405 | 131_S_0436 | 941_S_1202 |
| 011_S_0326 | 021_S_0231 | 031_S_0294 | 041_S_1425 | 098_S_0667 | 126_S_0506 | 131_S_1389 | 941_S_1203 |
| Table S2 List of participants selected from the ADNI dataset for validating the ordinal regression model. | | | | | | | |
