# Supplementary material for: Predicting Progression of Alzheimer’s Disease Using Ordinal Regression
Source: PLoS One. 2014 Aug 20;9(8):e105542. doi: 10.1371/journal.pone.0105542 (PMC4139338; doi:10.1371/journal.pone.0105542)
Supplement: Table S3 — Variables included in the ordinal regression analysis. 57 variables in total, 34 cortical thickness measures and 23 volumetric measures. (DOCX) [file pone.0105542.s005.docx]

| **Cortical thickness measures** | **Volumetric measures** |
| --- | --- |
| Banks of superior temporal sulcus | Third ventricle |
| Caudal anterior cingulate | Fourth ventricle |
| Caudal middle frontal gyrus | Brainstem |
| Cuneus cortex | Corpus callosum anterior |
| Entorhinal cortex | Corpus callosum central |
| Fusiform gyrus | Corpus callosum midanterior |
| Inferior parietal cortex | Corpus callosum midposterior |
| Inferior temporal gyrus | Corpus callosum posterior |
| Isthmus of cingulate cortex | CSF |
| Lateral occipital cortex | Accumbens |
| Lateral orbitofronral cortex | Amygdala |
| Lingual gyrus | Caudate |
| Medial orbitalfrontal cortex | Cerebellum Cortex |
| Middle temporal gyrus | Cerebellum White Matter |
| Parahippocampal gyrus | Hippocampus |
| Paracentral sulcus | inferior lateral ventricle |
| Frontal operculum | Putamen |
| Orbital operculum | Cerebral Cortex |
| Triangular part of inferior frontal gyrus | Cerebral White Matter |
| Pericalcarine cortex | Lateral Ventricle |
| Postcentral gyrus | Pallidum |
| Posterior cingulate cortex | Thalamus Proper |
| Precentral gyrus | Ventral DC |
| Precuneus cortex |  |
| Rostral anterior cingulate cortex |  |
| Rostral middle frontal gyrus |  |
| Superior frontal gyrus |  |
| Superior parietal gyrus |  |
| Superior temporal gyrus |  |
| Supramarginal gyrus |  |
| Frontal pole |  |
| Temporal pole |  |
| Transverse temporal cortex |  |
| Insular |  |
| **Table S3.** Variables included in the ordinal regression analysis.  57 variables in total, 34 cortical thickness measures and 23 volumetric measures. | |
