# Supplementary material for: Predicting Progression of Alzheimer’s Disease Using Ordinal Regression
Source: PLoS One. 2014 Aug 20;9(8):e105542. doi: 10.1371/journal.pone.0105542 (PMC4139338; doi:10.1371/journal.pone.0105542)
Supplement: Text S1 — (DOCX) [file pone.0105542.s006.docx]

***Supplementary Material for “A continuum-based measure of Alzheimer’s disease progression using ordinal regression.”***

**Methods**

*Study Information for AddNeuroMed and ADNI*

The AddNeuroMed project is part of the InnoMed European Union FP6 programme (Innovative Medicines in Europe), designed to develop and validate novel surrogate markers in Alzheimer’s disease (AD) [[1](#_ENREF_1)]. It includes a human neuroimaging strand which combines MRI data with other biomarkers and clinical data. Data was collected from six different sites across Europe: University of Kuopio, Finland; University of Perugia, Italy; Aristotle University of Thessaloniki, Greece; King’s College London, United Kingdom; University of **Ł**odz, Poland; and University of Toulouse, France. Written consent was obtained where the research participant had capacity, and in those cases where dementia compromised capacity then assent from the patient and written consent from a relative, according to local law and process, was obtained. This study was approved by ethical review boards in each participating country.

The ADNI dataset was downloaded from the Alzheimer’s Disease Neuroimaging Initiative database (www.loni.ucla.edu/ADNI, PI Michael M. Weiner). ADNI was launched in 2003 by the National Institute on Aging (NIA), the National Institute of Biomedical Imaging and Bioengineering (NIBIB), the Food and Drug Administration (FDA), private pharmaceutical companies and non-profit organizations, as a $60 million, 5-year public-private partnership. The primary goal of ADNI has been to test whether serial magnetic resonance imaging (MRI), of MCI and early AD. Determination of sensitive and specific markers of very early AD progression is intended to aid researchers and clinicians to develop new treatments and monitor their effectiveness, as well as lessen the time and cost of clinical trials. ADNI subjects aged 55 to 90 from over 50 sites across the U.S. and Canada participated in the research and more detailed information is available at [www.adni-info.org](http://www.adni-info.org/).

General inclusion and exclusion criteria for the studies were as follows.

For AddNeuroMed: **AD:** *Inclusion criteria*: ADRDA/NINCDS and DSM- IV (American Psychiatric Association 2000) criteria for probable AD, Mini Mental State Examination (MMSE) score range between 12 and 28, age 65 years or above. *Exclusion criteria*: significant neurological or psychiatric illness other than AD, significant unstable systematic illness or organ failure. All AD subjects had a Clinical Dementia Rating (CDR) scale score of 0.5 or above.

**CTL and MCI:** *Inclusion criteria*: MMSE score range between 24 and 30, Geriatric Depression Scale score less than or equal to 5, age 65 years or above, medication stable, good general health. *Exclusion criteria*: Meet the DSM- IV criteria for dementia, significant neurological or psychiatric illness other than AD, significant unstable systematic illness or organ failure. The distinction between MCI and controls was based on two criteria: CDR = 0 labelled the subject as control and a CDR = 0.5 labelled the subject as MCI. For the MCI subjects it was preferable that the subject and informant reported occurrence of memory problems.

CDR, Mini-Mental State, and CERAD Cognitive Battery were assessed for each subject. The CERAD Cognitive Battery was replaced with the Alzheimer's Disease Assessment Scale (ADAS–Cog) for the AD subjects. This cognitive test battery is specially designed for AD trials (Rosen *et al.* 1984). Both the ADAS-Cog and the CERAD battery use the same 10-word recall task, the only difference is that the scoring is inverted. The mean number of words not recalled in the CERAD word list immediate recall task was calculated. The variable obtained was named ADAS1, corresponding to the first subtest of ADAS-Cog. This was performed to have comparable measures for both the ADNI and AddNeuroMed cohorts.

For the ADNI cohort a detailed description of the inclusion criteria can be found on the ADNI webpage ([http://www.adni-info.org/Scientists/AboutADNI.aspx#](http://www.adni-info.org/Scientists/AboutADNI.aspx)). Subjects were between 55 and 90 years of age. They had a study partner able to provide an independent evaluation of functioning, and spoke either English or Spanish. All subjects were willing and able to undergo all test procedures including neuroimaging and agreed to longitudinal follow up. Specific psychoactive medications were excluded.

| 002_S_0295 | 011_S_1282 | 022_S_0129 | 032_S_0479 | 051_S_1072 | 082_S_1119 | 114_S_1106 | 130_S_0285 |
| --- | --- | --- | --- | --- | --- | --- | --- |
| 002_S_0413 | 012_S_0634 | 022_S_0130 | 032_S_0677 | 051_S_1123 | 082_S_1256 | 114_S_1118 | 130_S_0423 |
| 002_S_0559 | 012_S_0637 | 022_S_0543 | 032_S_0718 | 051_S_1131 | 082_S_1377 | 116_S_0361 | 130_S_0449 |
| 002_S_0619 | 012_S_0689 | 022_S_0544 | 032_S_1101 | 051_S_1296 | 094_S_0434 | 116_S_0370 | 130_S_0505 |
| 002_S_0685 | 012_S_0712 | 022_S_0961 | 032_S_1169 | 051_S_1331 | 094_S_0526 | 116_S_0382 | 130_S_0783 |
| 002_S_0729 | 012_S_0720 | 022_S_1097 | 033_S_0516 | 052_S_0671 | 094_S_0531 | 116_S_0392 | 130_S_0886 |
| 002_S_0782 | 012_S_0803 | 022_S_1394 | 033_S_0567 | 052_S_0951 | 094_S_0692 | 116_S_0487 | 130_S_0956 |
| 002_S_0816 | 012_S_0932 | 023_S_0031 | 033_S_0723 | 052_S_1054 | 094_S_0711 | 116_S_0648 | 130_S_0969 |
| 002_S_0938 | 012_S_1033 | 023_S_0042 | 033_S_0724 | 052_S_1250 | 094_S_0921 | 116_S_0657 | 130_S_1201 |
| 002_S_0954 | 012_S_1133 | 023_S_0058 | 033_S_0733 | 052_S_1251 | 094_S_1027 | 116_S_0752 | 130_S_1290 |
| 002_S_1018 | 012_S_1165 | 023_S_0061 | 033_S_0734 | 052_S_1346 | 094_S_1090 | 116_S_0834 | 130_S_1337 |
| 002_S_1155 | 012_S_1212 | 023_S_0078 | 033_S_0739 | 053_S_0389 | 094_S_1102 | 116_S_1232 | 131_S_0123 |
| 002_S_1261 | 012_S_1292 | 023_S_0081 | 033_S_0741 | 053_S_0507 | 094_S_1164 | 116_S_1249 | 131_S_0319 |
| 002_S_1268 | 012_S_1321 | 023_S_0083 | 033_S_0889 | 053_S_0621 | 094_S_1267 | 121_S_1322 | 131_S_0441 |
| 002_S_1280 | 013_S_0502 | 023_S_0084 | 033_S_0920 | 053_S_0919 | 094_S_1293 | 121_S_1350 | 131_S_0457 |
| 003_S_0907 | 013_S_0575 | 023_S_0093 | 033_S_0922 | 053_S_1044 | 094_S_1314 | 123_S_0072 | 131_S_0497 |
| 003_S_0908 | 013_S_0860 | 023_S_0139 | 033_S_0923 | 057_S_0464 | 094_S_1330 | 123_S_0088 | 131_S_0691 |
| 003_S_0931 | 013_S_0996 | 023_S_0376 | 033_S_1016 | 057_S_0474 | 094_S_1397 | 123_S_0091 | 131_S_1301 |
| 003_S_0981 | 013_S_1035 | 023_S_0855 | 033_S_1086 | 057_S_0643 | 094_S_1398 | 123_S_0094 | 132_S_0987 |
| 003_S_1074 | 013_S_1120 | 023_S_0916 | 033_S_1098 | 057_S_0818 | 094_S_1402 | 123_S_0106 | 133_S_0433 |
| 003_S_1122 | 013_S_1161 | 023_S_0926 | 033_S_1116 | 057_S_0934 | 094_S_1417 | 123_S_0113 | 133_S_0488 |
| 005_S_0221 | 013_S_1186 | 023_S_0963 | 033_S_1279 | 057_S_0941 | 098_S_0149 | 123_S_0162 | 133_S_0493 |
| 005_S_0222 | 013_S_1205 | 023_S_1046 | 033_S_1281 | 057_S_1217 | 098_S_0160 | 123_S_0298 | 133_S_0525 |
| 005_S_0324 | 013_S_1275 | 023_S_1126 | 033_S_1284 | 057_S_1269 | 098_S_0171 | 123_S_1300 | 133_S_0629 |
| 005_S_0448 | 013_S_1276 | 023_S_1190 | 033_S_1285 | 057_S_1371 | 098_S_0269 | 126_S_0605 | 133_S_0638 |
| 005_S_0546 | 014_S_0169 | 023_S_1247 | 033_S_1308 | 057_S_1373 | 098_S_0896 | 126_S_0606 | 133_S_0771 |
| 005_S_0553 | 014_S_0328 | 023_S_1262 | 033_S_1309 | 062_S_0535 | 099_S_0040 | 126_S_0680 | 133_S_0792 |
| 005_S_0572 | 014_S_0519 | 024_S_0985 | 035_S_0048 | 062_S_0578 | 099_S_0051 | 126_S_0709 | 133_S_0912 |
| 005_S_0602 | 014_S_0520 | 024_S_1171 | 035_S_0156 | 062_S_0690 | 099_S_0054 | 126_S_0784 | 133_S_0913 |
| 005_S_0610 | 014_S_0557 | 024_S_1307 | 035_S_0204 | 062_S_0730 | 099_S_0060 | 126_S_0891 | 133_S_1031 |
| 005_S_0814 | 014_S_0558 | 027_S_0074 | 035_S_0292 | 062_S_0768 | 099_S_0090 | 126_S_1187 | 133_S_1055 |
| 005_S_0929 | 014_S_0658 | 027_S_0116 | 035_S_0341 | 062_S_0793 | 099_S_0111 | 126_S_1221 | 133_S_1170 |
| 005_S_1224 | 014_S_1095 | 027_S_0118 | 035_S_0555 | 062_S_1099 | 099_S_0291 | 127_S_0259 | 136_S_0086 |
| 005_S_1341 | 016_S_0354 | 027_S_0120 | 036_S_0576 | 062_S_1182 | 099_S_0352 | 127_S_0260 | 136_S_0107 |
| 006_S_0498 | 016_S_0359 | 027_S_0256 | 036_S_0577 | 067_S_0019 | 099_S_0372 | 127_S_0393 | 136_S_0184 |
| 006_S_0547 | 016_S_0538 | 027_S_0307 | 036_S_0656 | 067_S_0029 | 099_S_0470 | 127_S_0394 | 136_S_0186 |
| 006_S_0681 | 016_S_0991 | 027_S_0403 | 036_S_0672 | 067_S_0038 | 099_S_0533 | 127_S_0431 | 136_S_0194 |
| 006_S_0731 | 016_S_1028 | 027_S_0404 | 036_S_0673 | 067_S_0056 | 099_S_0534 | 127_S_0622 | 136_S_0196 |
| 007_S_0068 | 016_S_1117 | 027_S_0408 | 036_S_0748 | 067_S_0059 | 099_S_1034 | 127_S_0684 | 136_S_0299 |
| 007_S_0070 | 018_S_0043 | 027_S_0417 | 036_S_0759 | 067_S_0076 | 099_S_1144 | 127_S_0754 | 136_S_0300 |
| 007_S_0249 | 018_S_0080 | 027_S_0461 | 036_S_0760 | 067_S_0077 | 100_S_0006 | 127_S_0844 | 136_S_0579 |
| 007_S_0316 | 018_S_0087 | 027_S_0485 | 036_S_0813 | 067_S_0110 | 100_S_0015 | 127_S_0925 | 136_S_0695 |
| 007_S_0344 | 018_S_0142 | 027_S_0644 | 036_S_0945 | 067_S_0176 | 100_S_0035 | 127_S_1140 | 136_S_1227 |
| 007_S_0414 | 018_S_0286 | 027_S_0850 | 036_S_1001 | 067_S_0177 | 100_S_0047 | 127_S_1419 | 137_S_0158 |
| 007_S_0698 | 018_S_0335 | 027_S_1045 | 036_S_1023 | 067_S_0243 | 100_S_0069 | 127_S_1427 | 137_S_0283 |
| 007_S_1206 | 018_S_0369 | 027_S_1081 | 037_S_0150 | 067_S_0257 | 100_S_0190 | 128_S_0135 | 137_S_0301 |
| 007_S_1222 | 018_S_0425 | 027_S_1082 | 037_S_0303 | 067_S_0284 | 100_S_0296 | 128_S_0200 | 137_S_0366 |
| 007_S_1304 | 018_S_0633 | 027_S_1213 | 037_S_0327 | 067_S_0290 | 100_S_0747 | 128_S_0216 | 137_S_0459 |
| 007_S_1339 | 018_S_0682 | 027_S_1254 | 037_S_0377 | 067_S_0336 | 100_S_0930 | 128_S_0225 | 137_S_0481 |
| 009_S_0751 | 020_S_0097 | 027_S_1385 | 037_S_0454 | 067_S_0607 | 100_S_0995 | 128_S_0229 | 137_S_0631 |
| 009_S_0842 | 020_S_0213 | 029_S_0824 | 037_S_0467 | 067_S_0812 | 100_S_1062 | 128_S_0272 | 137_S_0668 |
| 009_S_0862 | 020_S_1288 | 029_S_0836 | 037_S_0501 | 067_S_1253 | 100_S_1226 | 128_S_0310 | 137_S_0686 |
| 009_S_1030 | 021_S_0141 | 029_S_0866 | 037_S_0552 | 068_S_0109 | 100_S_1286 | 128_S_0500 | 137_S_0796 |
| 010_S_0067 | 021_S_0159 | 029_S_0914 | 037_S_0588 | 068_S_0127 | 109_S_0950 | 128_S_0517 | 137_S_0800 |
| 010_S_0419 | 021_S_0273 | 029_S_0999 | 037_S_0627 | 068_S_0210 | 109_S_0967 | 128_S_0522 | 137_S_0825 |
| 010_S_0420 | 021_S_0276 | 029_S_1056 | 037_S_1078 | 068_S_0442 | 109_S_1014 | 128_S_0528 | 137_S_0972 |
| 010_S_0472 | 021_S_0332 | 029_S_1215 | 037_S_1225 | 068_S_0473 | 109_S_1114 | 128_S_0545 | 137_S_0994 |
| 010_S_0786 | 021_S_0337 | 029_S_1218 | 037_S_1421 | 068_S_0872 | 109_S_1157 | 128_S_0608 | 137_S_1041 |
| 010_S_0829 | 021_S_0343 | 029_S_1318 | 041_S_0125 | 068_S_1075 | 109_S_1183 | 128_S_0715 | 137_S_1414 |
| 011_S_0002 | 021_S_0424 | 029_S_1384 | 041_S_0262 | 072_S_0315 | 109_S_1343 | 128_S_0740 | 141_S_0717 |
| 011_S_0005 | 021_S_0626 | 031_S_0321 | 041_S_0282 | 073_S_0089 | 114_S_0166 | 128_S_0770 | 141_S_0726 |
| 011_S_0008 | 021_S_0642 | 031_S_0351 | 041_S_0446 | 073_S_0311 | 114_S_0173 | 128_S_0863 | 141_S_0851 |
| 011_S_0010 | 021_S_0647 | 031_S_0554 | 041_S_0549 | 073_S_0312 | 114_S_0374 | 128_S_1088 | 141_S_0852 |
| 011_S_0016 | 021_S_0753 | 031_S_0568 | 041_S_0598 | 073_S_0386 | 114_S_0378 | 128_S_1242 | 141_S_0915 |
| 011_S_0021 | 021_S_0984 | 031_S_0618 | 041_S_0679 | 073_S_0565 | 114_S_0410 | 128_S_1406 | 141_S_0982 |
| 011_S_0023 | 021_S_1109 | 031_S_0830 | 041_S_1002 | 073_S_0746 | 114_S_0416 | 128_S_1409 | 141_S_1004 |
| 011_S_0053 | 022_S_0004 | 031_S_0867 | 041_S_1260 | 082_S_0304 | 114_S_0458 | 128_S_1430 | 141_S_1137 |
| 011_S_0183 | 022_S_0007 | 031_S_1066 | 041_S_1368 | 082_S_0363 | 114_S_0601 | 129_S_0778 | 141_S_1255 |
| 011_S_0241 | 022_S_0014 | 031_S_1209 | 041_S_1412 | 082_S_0832 | 114_S_0979 | 129_S_1246 | 941_S_1194 |
| 011_S_0856 | 022_S_0066 | 032_S_0147 | 041_S_1418 | 082_S_1079 | 114_S_1103 | 130_S_0102 | 941_S_1195 |
| 011_S_1080 | 022_S_0096 | 032_S_0400 | 041_S_1423 |  |  |  |  |
| Table S1 List of participants selected from the ADNI dataset for training and testing the ordinal regression model. | | | | | | | |

| 002_S_0955 | 011_S_0362 | 022_S_0219 | 032_S_0187 | 041_S_1435 | 098_S_0884 | 126_S_0708 | 133_S_0727 |
| --- | --- | --- | --- | --- | --- | --- | --- |
| 002_S_1070 | 011_S_0861 | 022_S_0750 | 032_S_0214 | 052_S_0952 | 099_S_0492 | 126_S_0865 | 136_S_0429 |
| 003_S_1021 | 012_S_1009 | 022_S_1351 | 032_S_0978 | 052_S_1352 | 100_S_0892 | 128_S_0138 | 136_S_0873 |
| 003_S_1057 | 013_S_0240 | 023_S_0126 | 032_S_1037 | 057_S_0779 | 100_S_1113 | 128_S_0167 | 136_S_0874 |
| 005_S_0223 | 013_S_0325 | 023_S_0217 | 033_S_0511 | 057_S_0839 | 109_S_0777 | 128_S_0188 | 137_S_0438 |
| 006_S_0484 | 013_S_0592 | 023_S_0331 | 033_S_0513 | 057_S_1007 | 109_S_0876 | 128_S_0227 | 137_S_0841 |
| 006_S_0653 | 014_S_0356 | 023_S_0388 | 033_S_0725 | 057_S_1265 | 109_S_1013 | 128_S_0230 | 137_S_0973 |
| 006_S_0675 | 014_S_0548 | 023_S_0604 | 033_S_0906 | 062_S_1299 | 109_S_1192 | 128_S_0245 | 141_S_0696 |
| 006_S_1130 | 014_S_0563 | 023_S_0887 | 035_S_0997 | 067_S_0098 | 114_S_0228 | 128_S_0258 | 141_S_0767 |
| 007_S_0041 | 016_S_0702 | 023_S_1289 | 036_S_0869 | 067_S_0828 | 116_S_0360 | 128_S_0611 | 141_S_0810 |
| 007_S_0101 | 016_S_0769 | 023_S_1306 | 036_S_0976 | 067_S_1185 | 116_S_0649 | 128_S_0947 | 141_S_0853 |
| 007_S_0128 | 016_S_1121 | 024_S_1063 | 036_S_1135 | 068_S_1191 | 116_S_1083 | 128_S_1043 | 141_S_1024 |
| 007_S_0293 | 016_S_1263 | 024_S_1393 | 036_S_1240 | 073_S_0518 | 116_S_1243 | 128_S_1148 | 141_S_1052 |
| 007_S_1248 | 018_S_0155 | 027_S_0179 | 037_S_0539 | 073_S_0909 | 116_S_1271 | 128_S_1408 | 141_S_1094 |
| 009_S_1334 | 018_S_0406 | 027_S_0835 | 037_S_0566 | 082_S_0640 | 116_S_1315 | 130_S_0232 | 141_S_1152 |
| 009_S_1354 | 018_S_0450 | 029_S_0843 | 041_S_0314 | 082_S_0761 | 123_S_0108 | 130_S_0289 | 141_S_1378 |
| 010_S_0904 | 020_S_0883 | 029_S_0878 | 041_S_0898 | 094_S_0489 | 123_S_0390 | 130_S_1200 | 941_S_1197 |
| 011_S_0022 | 020_S_0899 | 029_S_1073 | 041_S_1391 | 094_S_1241 | 126_S_0405 | 131_S_0436 | 941_S_1202 |
| 011_S_0326 | 021_S_0231 | 031_S_0294 | 041_S_1425 | 098_S_0667 | 126_S_0506 | 131_S_1389 | 941_S_1203 |
| Table S2 List of participants selected from the ADNI dataset for validating the ordinal regression model. | | | | | | | |

*MRI acquisition protocol*

Data acquisition for the AddNeuroMed study was designed to be compatible with the Alzheimer Disease Neuroimaging Initiative (ADNI) [[2](#_ENREF_2)]. The imaging protocol for both studies included a high resolution sagittal 3D T1-weighted MPRAGE volume (voxel size 1.1 x 1.1 x 1.2 mm^3^) and axial proton density / T2-weighted fast spin echo images. The MPRAGE volume was acquired using a custom pulse sequence specifically designed for the ADNI study to ensure compatibility across scanners [[2](#_ENREF_2)]. Full brain and skull coverage was required and a detailed quality control was carried out on all MR images according to the AddNeuroMed quality control procedure [[3](#_ENREF_3)].

| **Cortical thickness measures** | **Volumetric measures** |
| --- | --- |
| Banks of superior temporal sulcus | Third ventricle |
| Caudal anterior cingulate | Fourth ventricle |
| Caudal middle frontal gyrus | Brainstem |
| Cuneus cortex | Corpus callosum anterior |
| Entorhinal cortex | Corpus callosum central |
| Fusiform gyrus | Corpus callosum midanterior |
| Inferior parietal cortex | Corpus callosum midposterior |
| Inferior temporal gyrus | Corpus callosum posterior |
| Isthmus of cingulate cortex | CSF |
| Lateral occipital cortex | Accumbens |
| Lateral orbitofronral cortex | Amygdala |
| Lingual gyrus | Caudate |
| Medial orbitalfrontal cortex | Cerebellum Cortex |
| Middle temporal gyrus | Cerebellum White Matter |
| Parahippocampal gyrus | Hippocampus |
| Paracentral sulcus | inferior lateral ventricle |
| Frontal operculum | Putamen |
| Orbital operculum | Cerebral Cortex |
| Triangular part of inferior frontal gyrus | Cerebral White Matter |
| Pericalcarine cortex | Lateral Ventricle |
| Postcentral gyrus | Pallidum |
| Posterior cingulate cortex | Thalamus Proper |
| Precentral gyrus | Ventral DC |
| Precuneus cortex |  |
| Rostral anterior cingulate cortex |  |
| Rostral middle frontal gyrus |  |
| Superior frontal gyrus |  |
| Superior parietal gyrus |  |
| Superior temporal gyrus |  |
| Supramarginal gyrus |  |
| Frontal pole |  |
| Temporal pole |  |
| Transverse temporal cortex |  |
| Insular |  |
| **Table S3.** Variables included in the ordinal regression analysis.  57 variables in total, 34 cortical thickness measures and 23 volumetric measures. | |

*Gaussian process classification*

To compare the performance of ORGP to conventional binary classification approaches trained on CTL versus AD data Gaussian process classification (GPC) was employed . Similar to ORGP, learning is achieved using a Bayesian framework with a non-Gaussian likelihood. For information on GPC please refer to [[4](#_ENREF_4)] and for specific examples of the application of GPC to neuroimaging data please refer to [[5](#_ENREF_5), [6](#_ENREF_6)].

GP classifiers were trained on the CTL versus AD data and from the ADNI cohort and tested on the MCI-s/c data from the ADNI cohort and the MCI-s/c data from the AddNeuroMed cohort.

**Results**

*Binary classification using Gaussian process classification*

For the MCI-s/c subjects from the ADNI cohort a balanced accuracy of 66% with a sensitivity of 73% and a specificity of 60% were achieved. For the MCI-s/c subjects from the AddNeuroMed cohort a balanced accuracy of 69% with a sensitivity of 68% and a specificity of 70% were achieved. The confusion matrices for both cohorts are displayed in Figure S1.

| **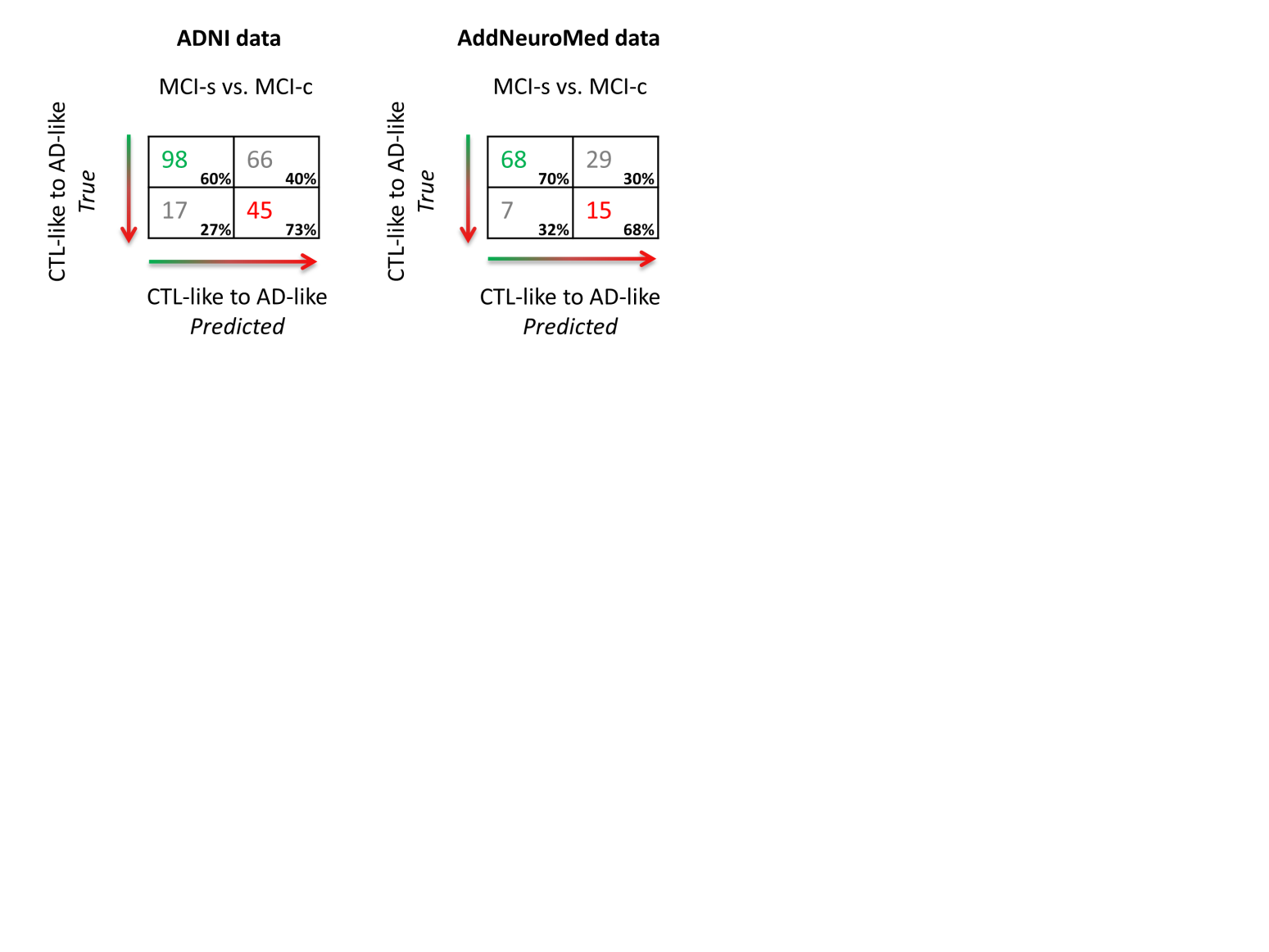** |
| --- |
| Figure S1 Confusion matrices obtained for MCI stable versus converters from the ADNI and AddNeuroMed cohorts using a binary Gaussian process classification trained on CTL versus AD subjects from the ADNI cohort. |

| 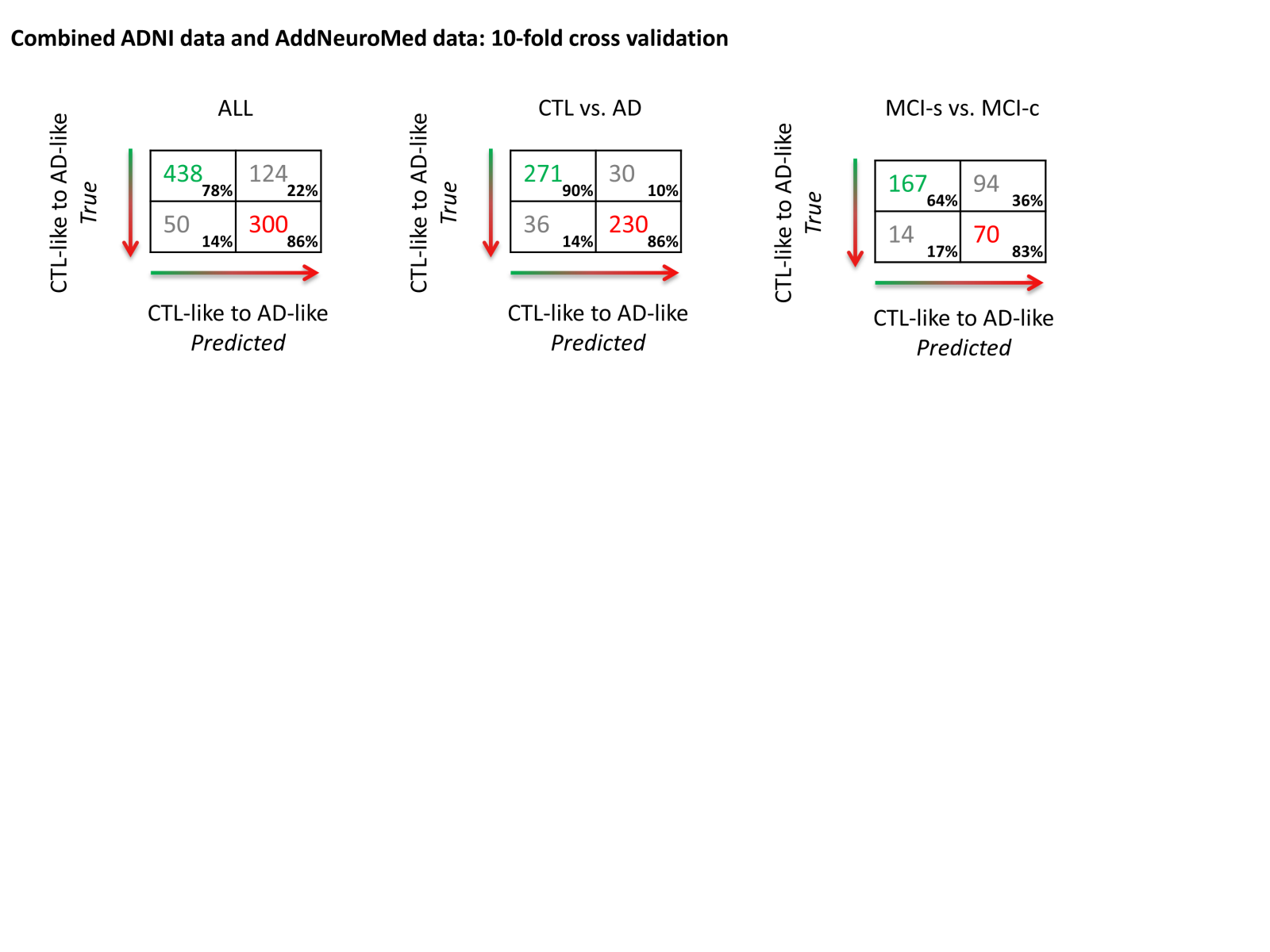 |
| --- |
| Figure S2 Confusion matrices for ordinal regression applied to the combined data from ADNI and AddNeuroMed using 10-fold cross validation. The confusion matrix for the binarised CTL-like vs. AD-like (CTL and MCI-s vs. MCI-c and AD) is displayed on the left. For illustration purposes, on the right confusion matrices for two contrasts of interest: CTL vs. AD and MCI-s vs. MCI-c (note: training scheme is unchanged). |

|  |
| --- |
|  |

**REFERENCES**

[1] S. Lovestone, P. Francis, and K. Strandgaard, "Biomarkers for disease modification trials--the innovative medicines initiative and AddNeuroMed," *J Nutr Health Aging,* vol. 11, pp. 359-61, Jul-Aug 2007.

[2] C. R. Jack, Jr., M. A. Bernstein, N. C. Fox, P. Thompson, G. Alexander, D. Harvey, B. Borowski, P. J. Britson, L. W. J, C. Ward, A. M. Dale, J. P. Felmlee, J. L. Gunter, D. L. Hill, R. Killiany, N. Schuff, S. Fox-Bosetti, C. Lin, C. Studholme, C. S. DeCarli, G. Krueger, H. A. Ward, G. J. Metzger, K. T. Scott, R. Mallozzi, D. Blezek, J. Levy, J. P. Debbins, A. S. Fleisher, M. Albert, R. Green, G. Bartzokis, G. Glover, J. Mugler, and M. W. Weiner, "The Alzheimer's Disease Neuroimaging Initiative (ADNI): MRI methods," *J Magn Reson Imaging,* vol. 27, pp. 685-91, Apr 2008.

[3] A. Simmons, Westman, E., Muehlboeck, S., Mecocci, P., Vellas, B., Tsolaki, M., Kloszewska, I., Wahlund, L-O., Soininen, H., Lovestone, S., Evans, A., Spenger C. for the AddNeuroMed consortium, "The AddNeuroMed framework for multi-centre MRI assessment of longitudinal changes in Alzheimer’s disease : experience from the first 24 months," *Int J Geriatr Psychiatry. 2011 Jan;26(1):75-82.,* 2011.

[4] C. E. Rasmussen and C. K. I. Williams. (2006). *Gaussian processes for machine learning* [Text].

[5] A. Marquand, M. Howard, M. Brammer, C. Chu, S. Coen, and J. Mourao-Miranda, "Quantitative prediction of subjective pain intensity from whole-brain fMRI data using Gaussian processes," *Neuroimage,* vol. 49, pp. 2178-2189, Feb 1 2010.

[6] O. M. Doyle, S. De Simoni, A. J. Schwarz, C. Brittain, O. G. O'Daly, S. C. R. Williams, and M. A. Mehta, "Quantifying the attenuation of the ketamine phMRI response in humans: a validation using antipsychotic and glutamatergic agents. ," *J Pharmacol Exp Ther,* vol. (In Press), 2013.
